# Pan-cancer multi-omics profiling of SRPK1 identifies tumour-specific associations with prognosis and predicted chemotherapy response in breast, colon and prostate cancer

**DOI:** 10.3389/fbinf.2026.1925354

**Published:** 2026-10-21

**Authors:** Duygu Duzgun, Sebastian Oltean

**Affiliations:** Department of Clinical and Biomedical Sciences, University of Exeter Medical School, Exeter, United Kingdom

**Keywords:** alternative splicing, chemoresistance, multi-omics, pan-cancer analysis, prognosis, SRPK1

## Abstract

**Purpose:**

Serine/arginine-rich protein kinase 1 (SRPK1), a regulator of splicing-factor activity and alternative splicing, has been implicated in cancer progression and therapy resistance. However, the tumour-specific expression landscape and clinical relevance of SRPK1 across human cancers remain incompletely defined. This study aimed to investigate SRPK1 expression, prognostic relevance, and associations with predicted chemotherapy response through pan-cancer multi-omics profiling, with a focused evaluation in breast, colon, and prostate cancer.

**Methods:**

Public transcriptomic, proteomic, immunohistochemical, survival, functional network and pharmacogenomic datasets were interrogated, including TCGA, GEPIA2, CPTAC/UALCAN, the Human Protein Atlas, GeneMANIA, STRING, GSCA and CPADS. SRPK1 mRNA and protein expression, survival associations, SRPK1-related splicing networks, SRPK1–SRSF1 co-expression and predicted drug-response associations were assessed in breast invasive carcinoma (BRCA), colon adenocarcinoma (COAD) and prostate adenocarcinoma (PRAD) cohorts.

**Results:**

SRPK1 displayed heterogeneous expression across TCGA tumour types, supporting tumour-specific dysregulation. SRPK1 mRNA and protein levels were increased in BRCA and COAD, whereas in PRAD, expression changes were more dataset-dependent. Survival analyses revealed cancer-type-dependent prognostic associations: high SRPK1 expression was associated with poorer outcomes in BRCA, whereas low SRPK1 expression was associated with poorer outcomes in COAD. SRPK1 was positively co-expressed with SRSF1 and mapped to splicing-related interaction networks. Pharmacogenomic analyses further linked SRPK1 expression to drug-sensitivity variation in cancer cell lines and to tumour-type-specific predicted chemotherapy response, including cisplatin in COAD and docetaxel in PRAD.

**Conclusion:**

These findings identify SRPK1 as a context-dependent molecular candidate associated with prognosis and predicted chemotherapy response in breast, colon and prostate cancer, supporting future experimental validation of SRPK1-linked splicing pathways in tumour-specific chemoresistance.

## 1 Introduction

As the second leading factor of mortality globally, cancer represents a significant burden on both current and future global health (Nagai and Kim, 2017). In 2022, there were about 20 million new cancer cases and 9.7 million cancer-related death cases, as published in the International Agency for Research on Cancer’s (IARC) GLOBOCAN report (Bray et al., 2024). By 2040, the number of cancer-related cases is projected to increase to approximately 28.4 million cases, an increase from 2022 by 42% (Brenner et al., 2023).

In these recent decades, there have been significant advancements in cancer biology research and the development of novel therapeutic approaches; however, the limitations of the treatment have made curing cancer a persevering challenge (Samantaray et al., 2024; Liu et al., 2024). The complex, dynamic, and adaptable nature of cancer must be comprehensively grasped for a more effective management of cancer. In cancer management, resistance to chemotherapy, the first-line treatment, is the biggest drawback of cancer treatment, posing an insurmountable problem (Cree and Charlton, 2017). Chemoresistance, which accounts for nearly 90% of treatment failures, poses a major obstacle in maximising treatment efficiency and is a leading cause of relapse and poor patient survival (Madukwe, 2023; Ramos et al., 2021). Despite extensive research in recent years, chemoresistance remains a critical limitation to the clinical application of commonly used chemotherapeutic agents. For instance, 5-fluorouracil is the primary treatment for patients with advanced colorectal cancer, but its antineoplastic efficacy is reduced due to the chemotherapeutic drug resistance of cancer cells through several interconnected mechanisms, including cytoproctective autophagy and the evasion of apoptosis, highlighting the multi-layered nature of chemoresistance (Li M. et al., 2024). Therefore, key mechanisms underlying chemoresistance reflect its profound biological complexity, which requires a multidimensional approach (Duzgun and Oltean, 2025).

Given the intricacies of chemoresistance, a pan-cancer expression analysis of any potential genes implicated in chemoresistance is necessary to examine their connection to clinical outcomes and explore the novel molecular mechanisms of chemoresistance. Such an approach potentially bolsters the effectiveness of cancer treatment, yielding more robust treatment responses and clinical outcomes.

### 1.1 A multi-omics approach in cancer research

High-throughput sequencing technologies have shown tremendous advancements and substantially revolutionised the overall environment and context of cancer research through their resilient and effective methodologies that yield novel possibilities in the study of molecular mechanisms associated with therapy resistance (Hutter and Zenklusen, 2018; Jiang et al., 2022). Central to this endeavour is the adoption of pan-cancer analysis, which is a robust integrative approach with the capacity to comparatively analyse genomic, epigenomic, transcriptomic, and proteomic data across a spectrum of cancer types (Campbell et al., 2020; Qian et al., 2020). There are clinical and multi-omics information from more than 20,000 specimens of 33 tumour types matched with normal tissues available in the Cancer Genome Atlas (TCGA) (Fonseca-Montaño et al., 2022; Avci et al., 2025). Analysis of such a rich dataset helps in establishing connections between gene expression characteristics in pretreatment tumour samples and identifying clinically relevant targets for more robust strategies to effectively deal with cancer resistance.

Recent studies have demonstrated that comparing multi-omics and experimental data can provide a unique opportunity for performing integrative analysis of therapy responders and non-responders (Yang et al., 2022; Vokes et al., 2022). In a multi-omics study on the role of solute carrier family 31 member 1 (SLC31A1), a key cisplatin transporter, in driving resistance to cisplatin, Qi et al. (2023) reported lower expression level of SLC31A1 in tumour tissues of renal cell carcinoma, lung adenocarcinoma, and prostate adenocarcinoma than that of their corresponding normal tissues, which was associated with reduced overall survival and progression-free survival in patients (Qi et al., 2023a). Additionally, zinc-finger protein 711 (ZNF711) has been reported to enhance SLC31A1 expression through histone methylation. The restored sensitivity to cisplatin in ovarian cancer cell lines through the overexpression of ZNF711 regulates SLC31A1, providing a specific target for cisplatin resistance (Wu et al., 2021). These experimental studies collectively demonstrated the integration of multi-omics data at the pan-cancer level as a robust approach for targeting drug resistance-associated genes and their underlying mechanisms, consequently laying the groundwork for future research on chemoresistance.

Despite numerous studies on this subject, the mechanisms underlying chemoresistance have remained underexplored, especially in cases that involve mutations, gene expression changes, alternative splicing, and post-translational protein modification due to the presence of complex and frequently multifactorial causes (Aleksakhina et al., 2019; Wu et al., 2021). With the recent RNA sequencing advancement, a growing body of evidence supporting the notion that drug resistance develops when cancer cells manage to subdue the cytotoxic effects associated with the administered chemotherapeutic agents by alternative splicing (Wu et al., 2021; Mehterov et al., 2021). Although research on this notion is still in its early stage, elucidating how key regulator-driven splicing networks converge on key pathways to drive chemoresistance yields valuable information on the therapeutic management of cancer. Building on this understanding, the analysis of multi-omics data to identify potential candidates that specifically target abnormal alternative splicing-driven chemoresistance paves the way for future cancer research on drug resistance.

### 1.2 Alternative splicing defects in cancer progression and chemoresistance

Alternative splicing (AS) is an essential post-transcriptional process that involves the expansion of the cell’s protein-coding repertoire through the generation of multiple distinct mature mRNAs by a single gene (Liu et al., 2017). Defects in splicing-regulatory elements and splicing factors, such as changes in expression levels or mutations, have been recently recognised as a hallmark of cancer (Sveen et al., 2016). These defects as the primary contributors to therapeutic resistance in numerous cancer types are presently explored (Duzgun and Oltean, 2025). Typical example includes the pyruvate kinase gene (PKM) splice variants that emerge under prolonged exposure to gemcitabine, resulting in the promotion of the PKM2 isoform through the selection of exon 10 (Wang et al., 2012; Zahra et al., 2020; Bai and Chen, 2020).

Based on a multi-omics database for the case of pancreatic ductal adenocarcinoma, a high level of PKM2 isoform was found to be correlated with shorter recurrence-free survival and poor prognosis patients (Calabretta et al., 2015). This splicing was subsequently shown to be guided by gemcitabine-induced upregulation of the polypyrimidine tract-binding protein (PTBP1), which serves as a major splicing factor that regulates PKM splicing, in chemoresistant pancreatic ductal adenocarcinoma cell lines. Reversing this splicing isoform towards the PKM1 isoform, which excludes exon 10, via knockdown of PTBP1, was able to restore sensitivity to gemcitabine in drug-resistant cells. Focusing on colon cancer (Cheng et al., 2018), also demonstrated enhanced sensitivity to vincristine and oxaliplatin in drug-resistant cancer cells through the regulation of glycolysis following the production of PKM1 isoform from silencing PTBP1 (Wu et al., 2021). These studies highlighted the promising prospects of PTBP1 as a therapeutic direction for drug-resistant cells resulting from splicing factor-induced AS, which has already been associated with patient clinical outcomes.

An integrative multi-omics analysis of clinical specimens prompts survival and differential analyses prior to experimental studies for the identification of novel potential targets linked to the development of drug resistance in cancer cells. This approach benefits future research on how to effectively overcome drug-resistance associated AS events in cancer cells.

### 1.3 The role of SRPK1 in chemoresistance

The discovery of the underlying causative roles and functional consequences of aberrant splicing is crucial to determine how splicing alterations contribute to drug resistance in cancer. Splicing decisions depend on various splicing factors phosphorylated by serine-arginine-protein kinases (SRPKs); hence, SRPKs have a critical role in regulating AS (Wu et al., 2021).

Studies have linked SRPK1, a key regulator of AS, and its downstream targets to a wide spectrum of malignancies, including breast, colon, lung, prostate, and retinoblastoma (Van Roosmalen et al., 2015; Mavrou et al., 2014; Yi et al., 2018; Gong et al., 2016; Krishnakumar et al., 2008; Patel et al., 2018). However, aberrant expression and functional alterations of SRPK1 appear to be related to both chemosensitivity and chemoresistance, raising an intriguing possibility on the role of SRPK1’s regulatory properties in stimulating the cellular response to anti-cancer drugs (Nikas et al., 2019). (Odunsi et al., 2012) observed restored sensitivity to cisplatin following the use of siRNAs to downregulate SRPK1, inhibiting ovarian cancer cell proliferation, migration, and invasion and promoting apoptosis (Krishnakumar et al., 2008). Conversely, Schenk et al. (2001) demonstrated reduced expression following the antisense oligonucleotide therapy targeting the translation initiation site of SRPK1, which enhanced ovarian cancer cell proliferation and cisplatin resistance (Krishnakumar et al., 2008). Hence, SRPK1 may be differentially modulated in distinct tumours; however, it remains largely unknown how tumour specific alterations contribute to chemoresistance mechanisms.

Wang et al. (2020); Huang et al. (2023) revealed the vital role of SRPK1 in cancer, which, if derailed, may result in the development of tumour resistance (Huang et al., 2023; Wang et al., 2020). However, the expression landscape and clinical relevance of SRPK1 across different tumour types have not been systematically investigated. In this article, we aimed to explore multi-omics data from diverse tumour types to establish a robust foundation for a more comprehensive and clinically comparative analysis of SRPK1 alterations. This analysis provides an important direction for further understanding of the tumour specific mechanisms by which SRPK1 contributes to the development of chemoresistance, highlighting its multifaceted roles in this context.

## 2 Materials and methods

### 2.1 Gene and protein expression analysis

Using the “Exploration—Gene_DE” module of Tumor Immune Estimation Resource 2.0 (TIMER2) web (Li et al., 2020) (http://timer.comp-genomics.org/), SRPK1 mRNA expression was assessed across TCGA tumour types by comparing primary tumour and adjacent normal tissues. Tumour-normal differences in SRPK1 mRNA expression were visualised using platform-generated box plots. Meanwhile, the “Expression Analysis—Expression DIY—Box Plot” function of GEPIA2 (Tang et al., 2019) (Gene Expression Profiling Interactive Analysis, version 2) web tool (http://gepia2.cancer-pku.cn/#analysis), which produced boxplots of SRPK1 expression differences between tumour and normal tissues, was utilised to complement the TIMER2 pan-cancer analysis by comparing TCGA tumour samples from breast invasive carcinoma (BRCA), colon adenocarcinoma (COAD) and prostate adenocarcinoma (PRAD) with the corresponding normal samples from the combined TCGA normal and GTEx datasets. For the GEPIA2 differential-expression analysis, expression values were log_2_(TPM +1)-transformed and tumour–normal differences were evaluated using one-way ANOVA. Benjamini–Hochberg false-discovery-rate adjustment was applied to the ANOVA-derived p-values, and differential expression was defined using *q < 0.01* together with |log2FC| (fold change) ≥ 1, where log_2_FC represents the difference between the median tumour and normal expression values. The |log_2_FC| ≥ 1 threshold corresponds to an approximately twofold difference on the TPM +1 scale and was retained as an effect-size criterion in addition to statistical significance.

At the protein level, Clinical Proteomic Tumor Analysis Consortium (CPTAC) proteomic datasets (Chen et al., 2019), accessed through the University of Alabama at Birmingham Cancer Data Analysis (UALCAN) portal (http://ualcan.path.uab.edu/analysis-prot.html), were utilised for the analysis of SRPK1 total-protein expression between primary tumour and normal tissues in BRCA and COAD, and between age-stratified primary-tumour groups in PRAD. Differences between groups were evaluated using a two-tailed unpaired Student’s t-test, with p < 0.05 considered statistically significant. In addition, the immunohistochemical assessment of SRPK1 protein expression (Antibody No. HPA016431) between normal and tumour tissues in BRCA, COAD, and PRAD was conducted qualitatively using the Human Protein Atlas (HPA) database (https://www.proteinatlas.org/).

### 2.2 Survival prognosis analysis

The overall survival (OS) and disease-free survival (DFS) significance map data of SRPK1 in BRCA, COAD, and PRAD were obtained using the “Expression Analysis—Survival Analysis—Survival Map” function of GEPIA2. Based on the median (50% cutoff-high and 50% cutoff-low) of SRPK1 expression, the clinical patient cases of BRCA, COAD, and PRAD were divided into high- and low-expression group. Statistical significance was assessed through the log-rank (Mantel–Cox) test, which involved determining the hazard ratio (HR) with a 95% confidence interval (CI). Besides that, the “Expression Analysis—Survival Analysis” function of GEPIA2 was utilised to determine the Kaplan-Meier plots of OS and DFS in BRCA, COAD, and PRAD. Using a Kaplan-Meier Plotter tool (https://kmplot.com/analysis/index.php?p=background), the relationship between patient prognosis and SRPK1 expression in BRCA and COAD (PRAD was excluded in this case due to data unavailability) was examined based on the generated Kaplan-Meier survival curves for OS and relapse-free survival (RFS or also called DFS). Patients were grouped into high- and low-expression groups for survival analysis based on the “Auto select best cutoff” function.

### 2.3 SRPK1-related genes and proteins enrichment analysis

Using the GeneMANIA (Warde-Farley et al., 2010) (https://genemania.org/) online portal, the function and association of the query gene SRPK1 in *Homo sapiens* were predicted for the construction of a gene–gene interaction network. The prediction output is in the form of a gene network that displays the top 20 interacting genes with SRPK1, where nodes symbolise genes and links represent networks.

A network analysis of SRPK1-binding proteins was subsequently searched on the STRING website (https://string-db.org/) (Szklarczyk et al., 2015) based on the following parameter settings: (1) the meaning of network edges was “confidence”; (2) the minimum required interaction score was “high confidence” (0.7); (3) the maximum number of interactions to show was “no more than 10 interactions” in the first shell. As a result, 10 proteins, including SRSF1, potentially interacted with SRPK1 were obtained. This study solely focused on SRSF1, a key regulator of alternative splicing, to evaluate its association with the survival information of patients with BRCA, COAD, and PRAD using the GEPIA2 and Kaplan-Meier Plotter databases, as described in Section 2.2.

Moreover, with the datasets of all TCGA tumours, the “Expression Analysis—Similar Gene Detection” function of GEPIA2 was utilised to assess the top 100 SRPK1-corrected targeting genes. Using the “Expression Analysis—Correlation Analysis” function of GEPIA2 in BRCA, COAD, and PRAD, Pearson’s correlation analysis of SRPK1 and the selected gene SRSF1 was then conducted. Through this module, dot plots were generated based on log2 [transcript per million (TPM)], yielding both *p-*values and correlation coefficients (R values).

### 2.4 Drug sensitivity analysis

The GSCA (https://guolab.wchscu.cn/GSCA/#/drug) pharmacogenomics platform (Liu et al., 2018), which comprises the integration of gene expression profiles for 33 cancer types from the TCGA, and drug sensitivity data from the GDSC (Genomics of Drug Sensitivity in Cancer) dataset (the half-maximal inhibitory concentrations (IC_50_) of 251 small molecules in 1,001 cell lines) and CTRP (Cancer Therapeutics Response Portal) dataset (the area under the dose-response curve (AUC) values of 481 small molecules in 960 cell lines) datasets of various small molecules across different cell lines and corresponding SRPK1 mRNA levels were utilised to assess the correlation between drug sensitivity and SRPK1 mRNA expression levels. In both GDSC and CTRP datasets, the association between SRPK1 mRNA expression and drug sensitivity was assessed using Pearson’s correlation coefficient and the corresponding false discovery rate (FDR)-adjusted values. Positive correlations were interpreted as associations between higher SRPK1 expression and relative drug resistance, whereas negative correlations were interpreted as associations between higher SRPK1 expression and relative drug sensitivity, in accordance with the GSCA drug-sensitivity output. For the top 30 drug-sensitivity associations displayed by GSCA, the platform-defined ranking procedure retained gene–drug pairs with an absolute correlation coefficient >0.1 and FDR <0.05 and ranked them using a score integrating the absolute correlation coefficient and −log_10_(FDR).

Subsequently, the drug responses (log(IC_50_)) for patient samples in the TCGA cohorts based on their SRPK1 expression profiles were predicted using the CPADS (Comprehensive Pancancer Analysis of Drug Sensitivity) pharmacogenomics platform (Li K. et al., 2024), which integrates gene expression profiles for 33 cancer types from the TCGA and corresponding drug sensitivity data from the GDSC dataset. As for the predicted drug responses, the “Drug Analysis—By Gene” function of CPADS was utilised to obtain predicted log(IC_50_) values for cisplatin in the BRCA and COAD patient cohorts and for docetaxel in the PRAD patient cohort. Among the agents for which predicted log(IC_50_) values were available in CPADS, cisplatin and docetaxel were selected because they represent two distinct mechanistic classes: docetaxel, a microtubule-targeting taxane that is a first-line agent in prostate cancer, and cisplatin, a DNA-damaging platinum compound representative of the class used in breast and colorectal cancer.

Clinical patient samples of BRCA, COAD, and PRAD were grouped into high- and low-expression groups based on the median cutoff value of SRPK1 expression. For that, the two-sided Wilcoxon rank-sum test was conducted to evaluate the differences in median −log10 (IC_50_) values of each selected drug between high- and low-expression groups (based on the threshold of *p* < 0.05).

### 2.5 Statistical analysis

The Wilcoxon rank-sum test was conducted to examine SRPK1 mRNA expression between tumour and normal tissues in TIMER2, whereas GEPIA2 tumour–normal differential-expression analyses were evaluated using one-way ANOVA with Benjamini–Hochberg FDR adjustment for multiple testing. Meanwhile, SRPK1 total-protein expression comparisons using CPTAC datasets accessed through UALCAN were evaluated using Student’s t-test (two-tailed, unpaired). Besides that, log-rank (Mantel–Cox) test and Kaplan-Meier Plotter were employed to assess the prognostic relevance of SRPK1 expression. Additionally, the correlations of SRPK1 with various clinical features, such as SRSF1 and drug sensitivity, were examined through Pearson’s correlation analysis. Unless otherwise specified for a platform-specific analysis, statistical significance was established at *p* < 0.05.

The multi-omics data resources and analytical tools used in the SRPK1 analysis workflow, including version/release or access-date information, key parameters, cohort-stratification approaches and random-seed applicability, are detailed in Supplementary Table S5.

### 2.6 Ethical approval and consent to participate

All data were obtained from publicly available databases that have already obtained ethical approval. Therefore, the analyses conducted in this study did not involve any ethical issues.

## 3 Results

An integrated multi-omics approach that took into account the unique SRPK1 expression profile of different tumour types and its drug sensitivity, functional interactions, and correlation with clinical prognostic value was utilised for this study’s pan-cancer analysis of SRPK1. Figure 1 delineates the comprehensive framework of this multi-omics approach.

**FIGURE 1:**
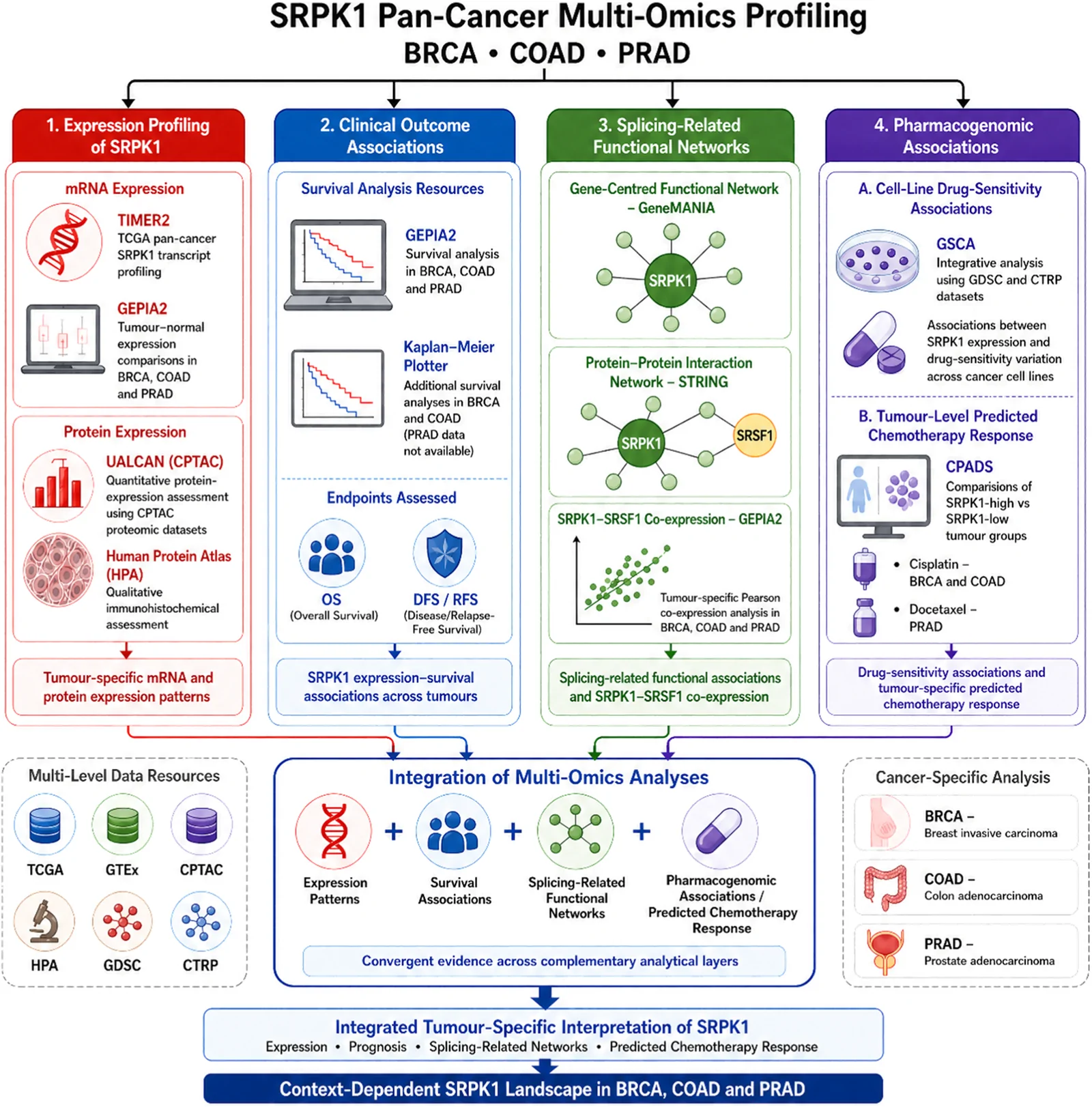
Summary of the study workflow and multi-omics databases used in SRPK1 analysis. This workflow illustrates the sequential steps of a comprehensive multi-omics analysis. The SRPK1 expression was first analysed at mRNA and protein levels, followed by the analysis on the relationship between survival probability and SRPK1 expression pattern, SRPK1-correlated genes and interacting proteins, and the impact of SRPK1 expression on drug sensitivity. Created with Illustrae.co.

### 3.1 Tumour-specific expression patterns of SRPK1 based on transcriptomic and proteomic clinical data in BRCA, COAD, and PRAD

Previous studies indicated that SRPK1 promotes tumorigenesis in various cancer types (Liu et al., 2021; Malvi et al., 2020). The important question of whether aberrant expression of SRPK1 influences clinical outcomes in TCGA dataset remains unresolved. Thus, this study employed the pan-cancer analytical approach to examine the mRNA expression patterns of SRPK1 in cancer patients using TIMER2 database.

As shown in Figure 2A, as compared with normal tissues, SRPK1 mRNA expression was markedly upregulated in primary tissues for BLCA, BRCA, CESC, CHOL, COAD, ESCA, HNSC, LIHC, LUAD, LUSC, READ, STAD, and UCEC but downregulated in KICH and KIRC. However, no significant differences were detected in SRPK1 expression between primary tumours and paired normal tissues for GBM, KIRP, PAAD, PRAD, and THCA. Considering the frequent dysregulation of SRPK1 across various cancer types at transcriptomic level, the analysis then focused on its associations with specifically BRCA, COAD, and PRAD to better understand tumour-specific expression patterns underlying cancer heterogeneity. The limited number of normal tissue samples in TIMER2 database led to the analysis of SRPK1 mRNA expression in BRCA, COAD, and PRAD through the GEPIA2 platform, where normal tissues were incorporated from the combined TCGA normal and GTEx datasets for more robust tumour-normal comparison. The analysis of GEPIA2 dataset revealed that BRCA and COAD achieved higher expression of SRPK1 in primary tumours, as compared with normal tissues, but this trend for PRAD achieved no statistical significance (*p* = 0.39; Figure 2B).

**FIGURE 2:**
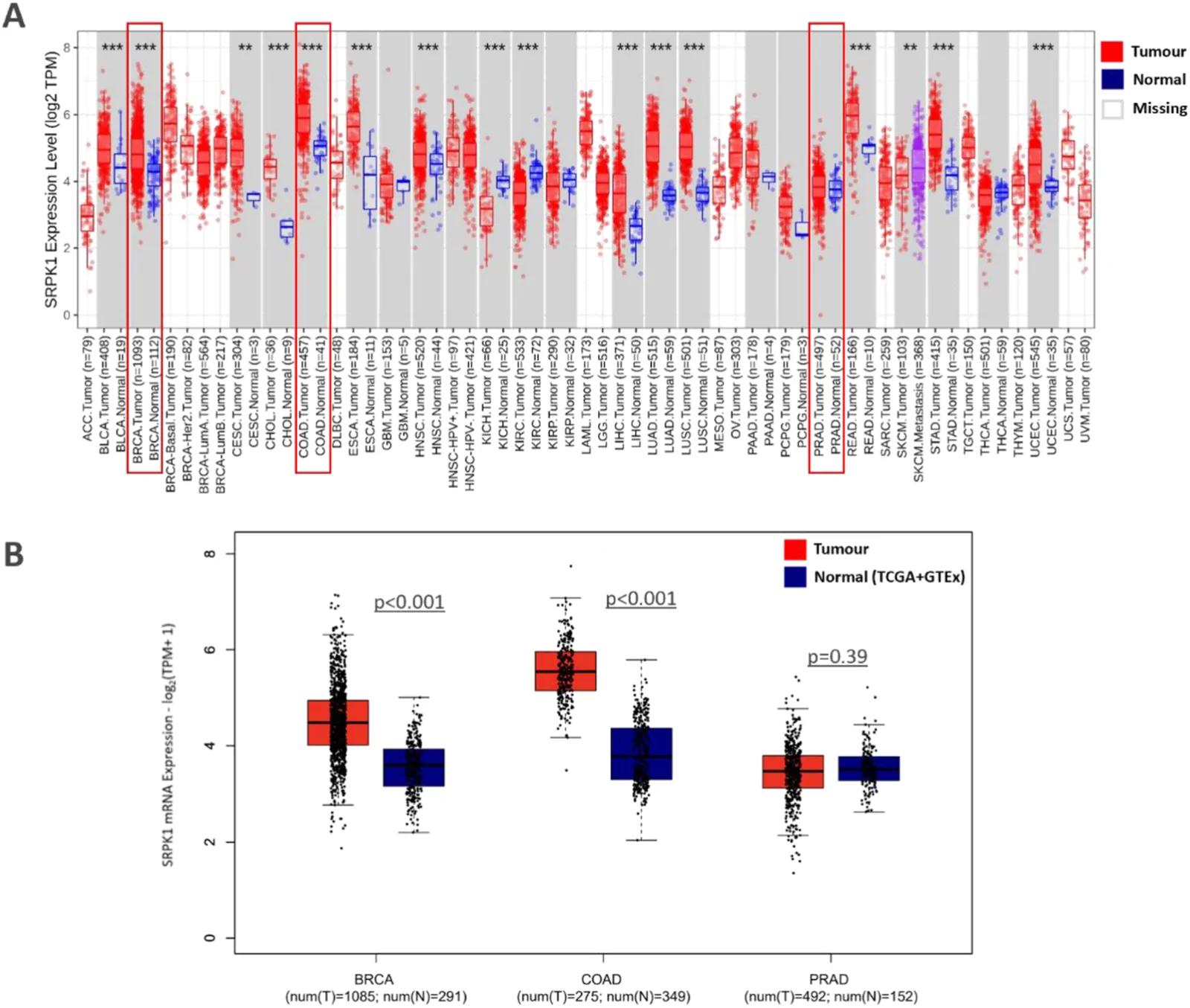
The differential mRNA expression of SRPK1 in pan-cancer samples **(A)** SRPK1 mRNA levels in different **tumour** and corresponding **normal** tissues, obtained from TIMER2 database. SRPK1 expression levels are given on the y-axis, calculated as log2 (TPM; transcripts per million). The primary tumour and adjacent normal tissues are presented along the x-axis, together in a **grey** area. The Wilcoxon rank-sum test was conducted to determine statistical significance: **denotes *p* < 0.01; *** denotes *p* < 0.001 **(B)** Analysis of SRPK1 mRNA expression levels in primary tumour tissues of patients with BRCA [num(T) = 1085], COAD [num(T) = 275], and PRAD [num(T) = 492] in the TCGA dataset, as compared with those in normal-matched tissues for breast [num(N) = 291], colon [num(N) = 349], and prostate [num(N) = 152] from the combined TCGA normal and GTEx datasets using the GEPIA2 platform. The transcriptional levels were log_2_(TPM +1)-transformed. Tumour–normal differential expression was evaluated using one-way ANOVA. Differential-expression classification was based on a Benjamini–Hochberg FDR-adjusted *q-value < 0.01* together with |log_2_FC| ≥ 1. The p-values displayed above the box plots represent the platform-reported tumour–normal comparison p-values. These graphs use **red** boxplots to denote primary tumour tissues and **blue** box plots to denote normal tissues.

In addition to the transcriptomic alterations of SRPK1, protein expression Clinical Proteomic Tumor Analysis Consortium (CPTAC) data quantified using the UALCAN platform were analysed. SRPK1 protein levels were compared between samples of primary tumour and paired adjacent normal tissues or age-stratified primary tumour tissues. In the case of primary tumour and adjacent normal tissues, the significantly higher SRPK1 protein levels in primary tumour samples in BRCA (*P* < 0.001; Figure 3A) and COAD (*P* < 0.001; Figure 3B) than their adjacent normal tissues were concordance with elevated mRNA levels for both BRCA and COAD. In the case of age-stratified primary tumour samples, PRAD patients aged 41–60 years and those aged 61–80 years recorded unchanged SRPK1 protein expression levels, possibly due to smaller sample sizes in loss of statistical power (*p* = 0.357; Figure 3C).

**FIGURE 3:**
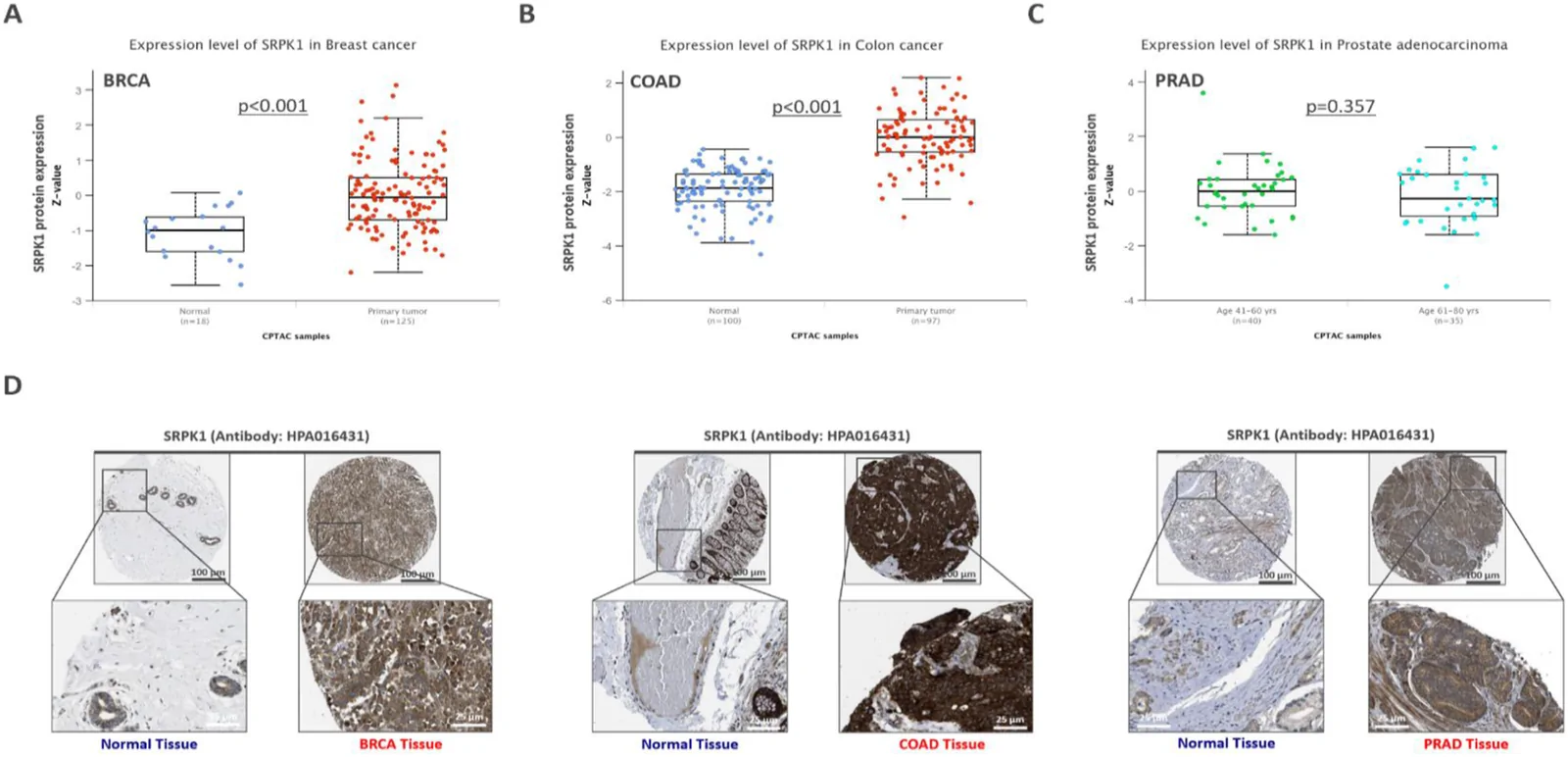
Differential expression analysis of SRPK1 protein levels in three different cancer tissues **(A,B)** Comparison of SRPK1 protein expression between primary tumour tissues of BRCA (in **red** ; n = 125) and COAD (in **red** ; n = 97) and their corresponding normal breast (in **blue** ; n = 18) and colon (in **blue** ; n = 100) tissues from the CPTAC dataset on the UALCAN portal **(C)** Analysis of the CPTAC-PRAD dataset for the protein expression of SRPK1 in age groups of 41–60 years (n = 40, highlighted in **green** ) and 61–80 years (n = 35, highlighted in **light blue** ). Z-values represent standard deviations from the median (the middle line in boxplot, with the lower and upper boundaries as the first and third quartiles and the whiskers extending to 1.5× the interquartile range of both quartiles) across samples for the given cancer types. Statistical significance (*p* < 0.05) of differential expression of SRPK1 was determined through an unpaired Student’s t-test; *n* represents the number of samples in each boxplot **(D)** Representative IHC images showing immunostaining for the SRPK1 protein in BRCA, COAD, and PRAD tissues and their corresponding normal tissues using HPA016431 antibody, obtained from the HPA dataset. Intense brown staining denotes increased expression in primary tumour tissues, as compared with normal tissues. Blue staining represents cell nuclei; brown staining shows SRPK1 protein deposits. Upper panel: scale bar = 100 μm; magnification 40×. Lower panel: scale bar = 25 μm; magnification 400×.

To further validate the protein levels of SRPK1 in those cancers, primary tumour tissues for which adjacent normal tissues were available were analysed using an alternative multi-omics HPA platform that included immunohistochemistry (IHC) staining data, where the degree of staining may more readily reflect protein levels. Based on the IHC staining of BRCA, COAD, and PRAD primary tissues for SRPK1, primary tumour tissues recorded significantly higher SRPK1 protein expression (mainly localised in the cytoplasm), as compared with the adjacent normal tissues (Figure 3D).

Taken together, clinical information and tissue samples were uneven across the many different pan-cancer types, but expression-based analysis revealed tumour-specific SRPK1 alterations, highlighting a potentiall role of SRPK1 in tumour progression. Supplementary Table S1 lists the abbreviation of each cancer type.

### 3.2 Cross-platform identification of gene-expression-defined prognostic features of SRPK1 in BRCA, COAD, and PRAD

Gene expression level associated to poor patient prognosis is indicative of that gene as a key contributor to the cancer progression (Kaelin, 2017) based on the assumption that, due to their conjectured role as cancer drivers in tumour-specific dependencies, prioritising gene expression changes associated with shorter survival times makes ideal therapeutic targets. In the following gene expression analysis, SRPK1 expression changes were identified as a significant adverse feature. Therefore, the analysis ensued by assessing whether these alterations could also be associated with poor prognosis in patient cohorts. GEPIA2 and Kaplan-Meier Plotter databases were utilised for the systematic analysis of the prognostic significance of SRPK1 expression status in BRCA, COAD, and PRAD cohorts. In the GEPIA2 database, the prognostic potential of SRPK1 was measured with a median cutoff point determined with HR for OS and DFS (in SRPK1-high-group *versus* SRPK1-low group for each cohort) (Figure 4).

**FIGURE 4:**
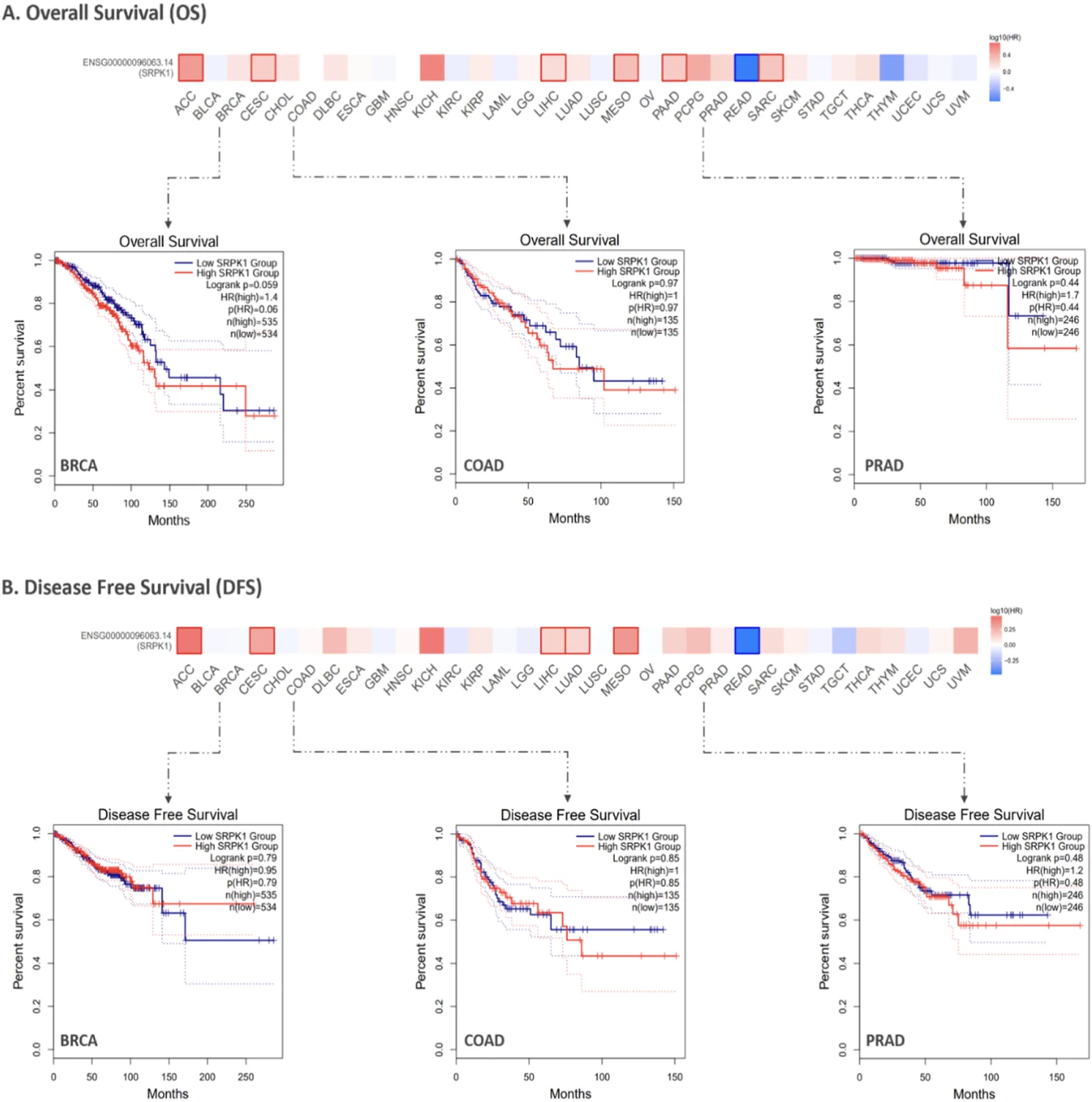
Survival analysis of patients with different SRPK1 mRNA expressions in BRCA, COAD, and PRAD clinical cohorts **(A)** OS and **(B)** DFS for BRCA, COAD, and PRAD patients were analysed using the GEPIA2 database. Patients in the indicated cancer cohorts were stratified based on the median cutoff of SRPK1 mRNA expression, yielding Kaplan-Meier curves of OS and DFS (plotted in months). The **red** and **blue** lines represent the survival curves of patients with high and low levels of SRPK1 mRNA. Indicated by dashed lines, HR values (with 95% confidence intervals) represent the relative risk of the low-expression group compared with the high-expression group. HR > 1 implies the gene as a potential risk factor, whereas HR < 1 suggests a protective factor. Based on a log-rank (Mantel-Cox) test, a log-rank *p* < 0.05 indicates statistical significance. *n* indicates the patient count in each expression group.

Patients with low SRPK1 (n = 534) recorded a nearly significant longer OS than those with high SRPK1 (n = 535) in the BRCA dataset under the median cutoff point (HR = 1.4; log-rank *p* = 0.059; Figure 4A, left). In the COAD (HR = 1; log-rank *p* = 0.97; Figure 4A, middle) and PRAD (HR = 1.7; log-rank *p* = 0.44; Figure 4A, right) datasets, with 135 and 246 patients respectively, a better OS was observed in patients with high SRPK1 than those with low SRPK1 despite the statistically insignificant differences.

Following that, a DFS analysis of all three cohorts was conducted, which revealed a trend towards high expression of SRPK1 being associated with disease progression in the BRCA dataset (HR = 0.95; log-rank *p* = 0.79; Figure 4B, left). As for the COAD (HR = 1; log-rank *p* = 0.85; Figure 4B, middle) and PRAD (HR = 1.2; log-rank *p* = 0.48; Figure 4B, right) datasets, high expression of SRPK1 was not associated with adverse outcomes. However, these associations were not statistically significant, which may be attributed to the small sample size of patients in clinical settings.

Therefore, this study considered the possibility that the utilisation of other related databases with larger datasets associated with BRCA, COAD, and PRAD patient survival outcomes may uncover prognostic relationships not found in this study’s GEPIA2 analysis. Addressing that, this study proceeded with an additional prognostic analysis using the Kaplan-Meier Plotter database, which contains larger patient datasets, to evaluate survival-associated gene expression patterns at the mRNA level. For Kaplan-Meier OS and DFS analyses, an optimal clinically relevant cutoff was applied. Patients were dichotomised into SRPK1-high and -low groups in the BRCA and COAD datasets (Figure 5) based on maximally selected log-rank statistics yielding hazard ratios. Please note that, in this database, corresponding data were not available for PRAD.

**FIGURE 5:**
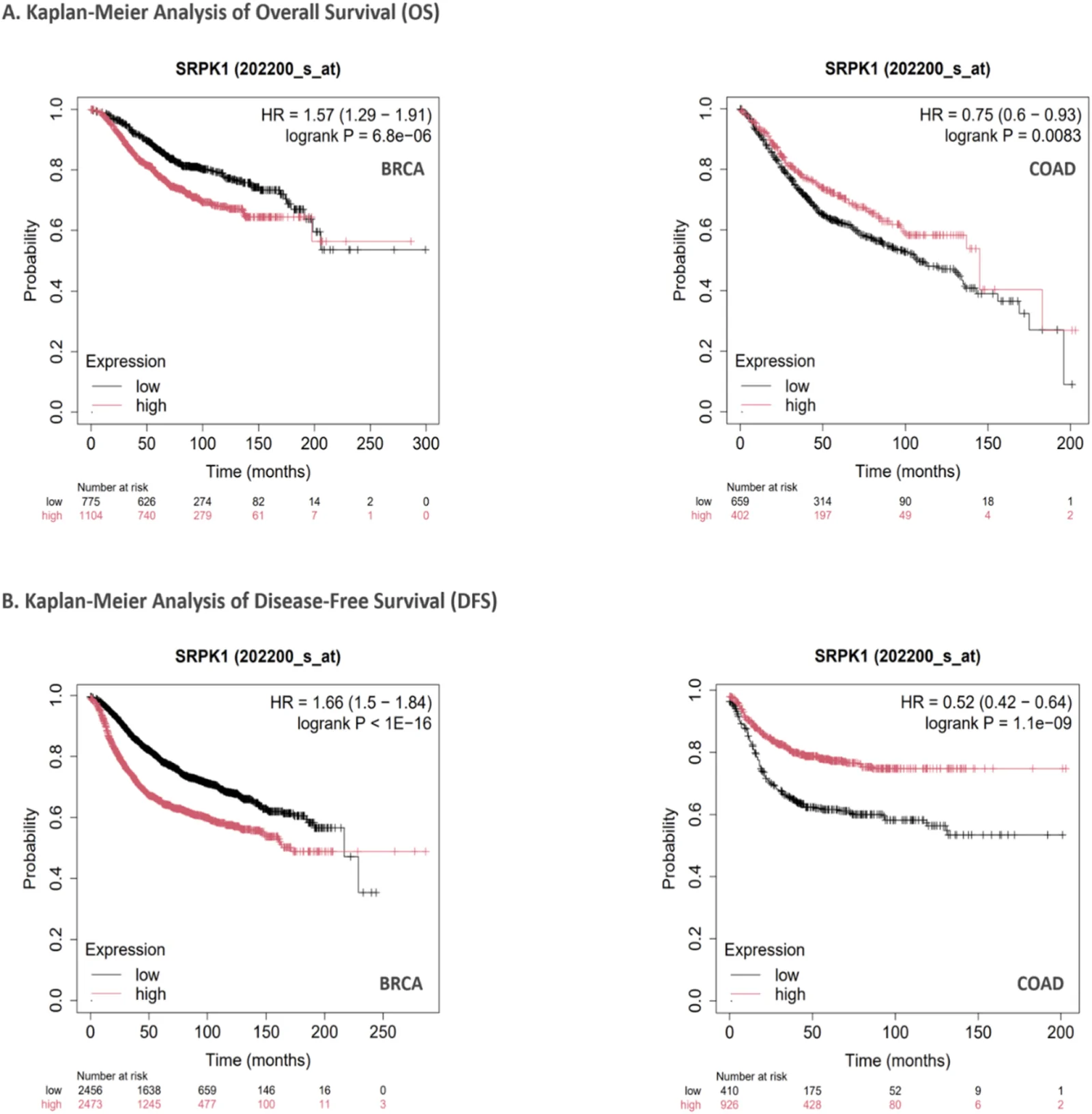
Analysis of the expression and prognosis relevance of SRPK1 in BRCA and COAD cohorts. Kaplan-Meier plots revealed differences in **(A)** OS and **(B)** DFS of BRCA and COAD patients based on the 202200_s_at probe obtained from the Kaplan-Meier plotter database. The OS and DFS of BRCA and COAD patients stratified by low ( **black** line) and high ( **red** line) SRPK1 expressions using the optimal cutoff point provided by Kaplan-Meier plotter database. The OS and DFS of high- and low-expression groups were compared based on the HR (with 95% confidence intervals). Log-rank (Mantel–Cox) test was performed to determine statistical significance; *p* < 0.05 indicates statistical significance.

Results of Kaplan-Meier survival analysis revealed significantly worse OS in BRCA patients with high SRPK1 expression (n = 1104) than those with low SRPK1 expression (n = 775) (HR = 1.57; log-rank *p* = 6.8e-06; Figure 5A, left). A similar trend was observed for DFS (high SRPK1: n = 2473, low SRPK1: n = 2456; HR = 1.66; log-rank *p* < 1E-16; Figure 5B, left). This suggested the potential pro-tumour role of upregulated SRPK1 in breast carcinogenesis. Meanwhile, low SRPK1 expression was found to be correlated with a marked decrease in OS (high SRPK1: n = 402, low SRPK1: n = 659; HR = 0.75; log-rank *p* = 0.0083; Figure 5A, right) and DFS (high SRPK1: n = 926, low SRPK1: n = 410; HR = 0.52; log-rank *p* = 1.1e-09; Figure 5B, right) in patients with COAD. This indicated that downregulation of SRPK1 may contribute to the progression of colon cancer. Collectively, this disparity reflected the complex and multifaceted role of SRPK1 expression in mediating cancer-type-dependent associations between clinical variables and poor outcomes.

### 3.3 Identification of gene–protein interactions associated with SRPK1-Bound functional networks underpinning alternative splicing

Cancer cells exhibit extensive heterogeneity in their cell states, characterised by altered gene and protein expression signatures associated with epithelial-to-mesenchymal transition (EMT), invasion, metastasis, aberrant RNA splicing, and therapeutic resistance, among other oncogenic processes (Marusyk et al., 2020; Patel and Yanai, 2024). This study’s preceding integrative multi-omics clinical data analyses suggested that the abnormal expression of SRPK1 mRNA and protein is broadly associated with unfavourable outcomes in a tumour-type-specific manner. This prompted this study to explore the functional network of genes and proteins interacting with SRPK1, specifically on whether the identification of gene–gene and protein–protein interaction networks could provide deeper insights on the SRPK1-dependent biological functions.

In order to identify gene(s) closely related to the regulatory functional network in which SRPK1 was involved, this study first performed a gene–gene interaction analysis using the GeneMANIA database on the top 20 genes most correlated with SRPK1. These genes were categorised as pan-cancer genes and exhibited a number of gene–gene interactions, which formed a densely interconnected ‘‘main’’ physical network, reflecting potential functional associations among SRPK1-bound genes shown in Figure 6A. Functional gene–gene interaction analysis across pan-cancer tumour samples revealed 20 SRPK1-related genes enriched in numerous cellular processes, including mRNA splicing and processing (*ZRSR2*, *RBM4*, *SRSF1*, *SF3A3*, *SRSF6*, *PRPF8*, *PRPF38A*, *TARDBP*), chromatin organisation and transcriptional regulation (*LBR*, *SAFB*, *SAFB2*, *NAT10*, *SIRT7*), ubiquitin-mediated proteolysis and protein homeostasis (*HECW2*, *PPM1G*), DNA damage response and cell cycle regulation (*GTF3C4*, *GBP2*), chromatin remodelling in germ cell development (*PRM1*), and immune response and signal transduction (*GBP2*, *SERPINB10*). These processes are crucial in therapeutic resistance (Pan et al., 2023; Xie et al., 2023; Sheng et al., 2018), tumour development, and progression (Ren et al., 2022; Yi et al., 2023; López-Cánovas et al., 2023).

**FIGURE 6:**
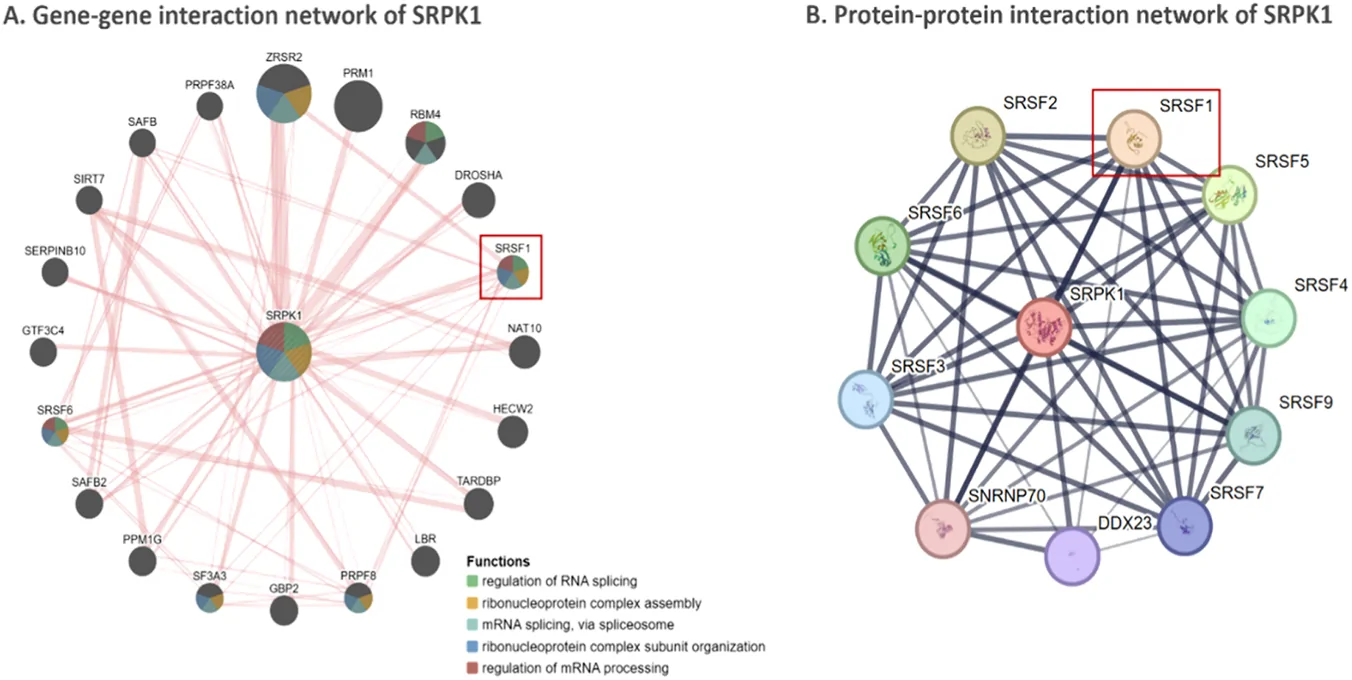
Functional enrichment analysis of SRPK1-interacting genes and proteins across pan-cancers **(A)** Gene–gene interaction network of SRPK1 obtained from the GeneMANIA database illustrates its physical and functional partners. In the network, genes are represented by **dark-grey** nodes, and their interactions are denoted by **pink** lines between nodes (edges). A node is represented by a pie chart that indicates the distribution of each gene’s functional categories associated with SRPK1. Pie chart colours: **green** denotes regulation of RNA splicing; **yellow** denotes ribonucleoprotein complex assembly; **aqua** denotes mRNA splicing via spliceosome; **blue** denotes ribonucleoprotein complex subunit organisation; **red** denotes regulation of mRNA processing. The selected gene, SRSF1, which exhibited the strongest functional association with SRPK1 among the interacting genes, is framed in a **red** square. Colour coding of the pie chart pieces is shown on the right side of the figure. **(B)** Protein–protein interaction network of SRPK1, generated using the STRING physical interactions database, identifies functional interactions associated with the pre-mRNA splicing process in tumours. Each node (coloured circle) represents a group of SRPK1-interaction proteins with functional relationships, and a **black** edge indicates an interaction. Node colours do not have any meaning. Protein–protein interactions were identified at a high confidence score (≥ 0.7). The thickness of the edges reflects the strength of supporting evidence, where thicker edges indicate higher interaction scores. The selected protein, SRSF1, outlined in a **red** rectangle, represents the strongest predicted interaction.

Next, a protein–protein interaction analysis was conducted using the STRING database based solely on experimentally validated sources to identify key proteins functionally interacting with SRPK1 across different cancer types (Figure 6B). This analysis revealed that a network consisting of serine/arginine-rich (SR) splicing factors, SRSF1, SRSF5, SRSF4, SRSF9, SRSF7, SRSF3, SRSF6, and SRSF2, the spliceosomal RNA helicase DDX23, and the U1 snRNP component SNRNP70, which strongly interacted with SRPK1, may mediate drug response by regulating the alternative splicing network. E2F1 transcriptionally activated DDX23, a splicing factor, which regulated the mRNA processing of FOXM1, promoting the progression of ovarian cancer progression (Zhao et al., 2021). The overexpression of SNRNP70, a nuclear RNA-binding protein regulating splice variants of transcripts, may drive the formation and growth of osteosarcoma by regulating the skipping of exon 10 of CD55 (Li W. et al., 2024). Among them, serine/arginine-rich (SR) family members—well-established drivers of tumorigenesis—particularly SRSF1, were highlighted, and their abnormal expression in various cancer types pointed to chemoresistance or drug-tolerance mechanisms by producing impaired alternative splicing patterns (Pan et al., 2023; Z et al., 2021; Pellarin et al., 2020; Bian et al., 2024; Wang et al., 2022; Sinnakannu et al., 2020).

Taken together, analysing these databases revealed that, beyond the growing recognition of the importance of abnormal RNA splicing in tumorigenesis, the identification of its gene–gene and protein–protein interaction networks captures a functional insight, revealing the potential role of SRPK1 as a contributor to therapeutic resistance. The description of gene functions is listed in Supplementary Table S2.

### 3.4 Co-expression of the selected splicing factor SRSF1 with SRPK1 and association with its prognostic significance in BRCA, COAD, and PRAD

Previous studies demonstrated that SRPK1 and SRPK1-associated splicing regulators are often aberrantly expressed in cancers and can drastically affect cancer malignancy through both the splicing-regulatory network and, at least in part, effective co-expression interactions with one another (Liu et al., 2021; Wagner et al., 2019). There are sporadic findings describing alterations in the expression and corresponding functions of these splicing regulatory elements in different malignancies. Nevertheless, clinically relevant data on their gene-level correlations in the context of their overexpression or downregulation should assist in dissecting their cancer-specific actions.

As the first step towards this, this study focused on splicing factor SRSF1, a prototypical SR family member, among the identified SRPK1-related genes/proteins (Figure 7). Apart from being broadly expressed in distinct cell types, it regulates the splicing pattern of many important genes (Das and Krainer, 2014). As for the dissection of the SRPK1-SRSF1 interaction at the mRNA level, their co-expression patterns in BRCA, COAD, and PRAD patient cohorts were analysed using the GEPIA2 database. Correlation analysis for COAD (*R* = 0.63; *p* < 0.001; Figure 7B) and PRAD (*R* = 0.68; *p* < 0.001; Figure 7C) patient samples revealed a strong positive correlation between SRPK1 and SRSF1 mRNA expression levels. The same results were obtained in BRCA patient samples (*R* = 0.45; *P* < 0.001; Figure 7A), but its correlation was moderate, as compared with that of COAD and PRAD patient samples. Results suggested the potential influence of SRSF1 on SRPK1-driven AS via a combination of mechanisms when co-expressed with SRPK1 in the analysed cancer types.

**FIGURE 7:**
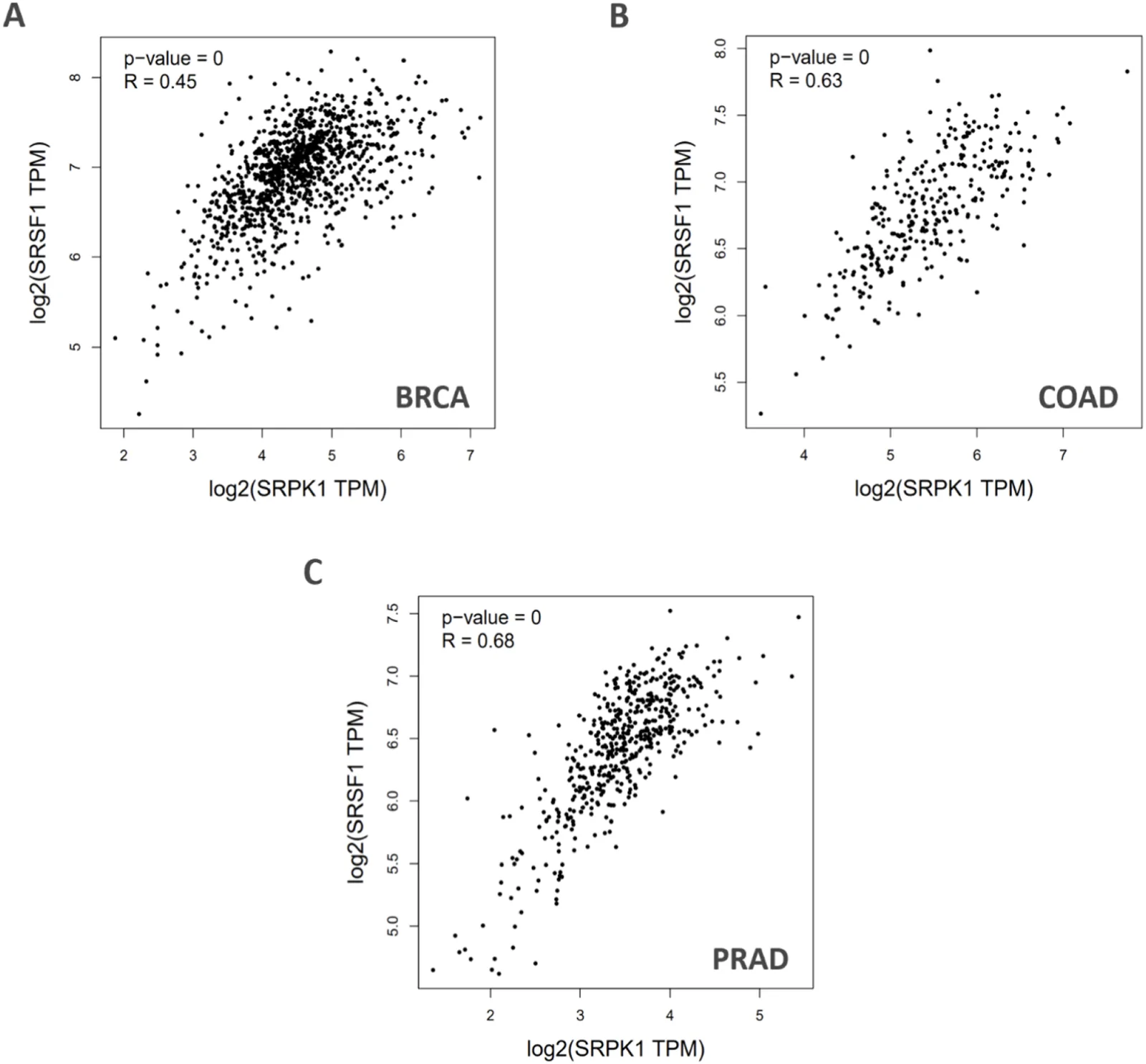
Selected splicing factor SRSF1 positively correlated with SRPK1 in BRCA, COAD, and PRAD patient samples. Scatterplots exhibit the correlation between SRPK1 (x-axis) and SRSF1 (y-axis) expression at the mRNA level based on **(A)** TCGA-BRCA **(B)** TCGA-COAD, and **(C)** TCGA-PRAD datasets analysed using the GEPIA2 database. The correlation coefficient (R value) was calculated using Pearson’s correlation, and the corresponding *p-*value indicates the statistical significance of the correlation. Each **black** dot represents the log2-transformed expression levels (TPM; transcripts per million) of both genes in an individual patient.

Meanwhile, the survival analysis of SRPK1 revealed the expression level of SRPK1, with significant association with poor outcome in cancer-type specific survival, as the most prominent prognostic feature. Given its predominant positive correlation with SRSF1, this study proceeded to examining the prognostic significance of SRSF1 expression in BRCA, COAD, and PRAD patient cohorts using two independent databases, namely, GEPIA2 and Kaplan-Meier Plotter databases.

This study first used the GEPIA2 database to assess the alterations in SRSF1 mRNA expression in relation to OS and DFS for BRCA, COAD, and PRAD patients, where tumour samples were categorised into high- and low-expression subgroups based on the median SRSF1 expression level in each patient cohort (as the cutoff value) (Supplementary Figure S1). The survival analysis demonstrated that high levels of SRSF1 tended to coincide with shorter OS in both BRCA (HR = 1.1; log-rank *p* = 0.49; Supplementary Figure S1A, left) and PRAD (HR = 1.8; log-rank *p* = 0.37; Supplementary Figure S1A, right) patient cohorts (n = 1,070 and n = 492, respectively) despite the statistical insignificance. In contrast, the data of the COAD patient cohort yielded a substantially inferior OS (HR = 0.55; log-rank *p* = 0.015; Supplementary Figure S1A, middle) for patients with SRSF1-low expression tumours (n = 135) compared with what was seen in those with SRSF1-high expression tumours (n = 135) although the number of tumour samples in both expression subgroups was rather low. Moreover, based on the results of DFS analyses, PRAD patients with SRSF1-low expression tumours tended to have better survival outcomes (n = 492; HR = 1; log-rank *p* = 0.83; Supplementary Figure S1B, right), whereas BRCA patients with SRSF1-low expression tumours tended to have worse clinical survival outcomes (n = 1,070; HR = 0.89; log-rank *p* = 0.55; Supplementary Figure S1B, left). A similar pattern to the BRCA patient cohort was observed in the COAD patient cohort (n = 270; HR = 0.72; log-rank *p* = 0.17; Supplementary Figure S1B, middle).

Overall, the difference in DFS between SRSF1-high expression group and SRSF1-low expression tumour group was not statistically significant. When analysing separately within the BRCA, COAD, and PRAD cohorts, most of the associations became insignificant, likely due to the relatively small sample size in each expression subgroup.

However, the small sample size involved, especially in COAD and PRAD patient cohorts, may obscure the prognostic effects associated with SRSF1 expression levels in the above analysis. With that, this study examined the associations of SRSF1 mRNA expression levels with OS and DFS by leveraging the large patient cohorts whose survival times were available in the Kaplan-Meier Plotter database. Tumour samples from BRCA and COAD cohorts were divided into high- and low-expression subgroups according to the optimal cutoff point. However, OS and DFS could not be assessed due to the unavailability of data for the PRAD cohort in the Kaplan-Meier Plotter database (Supplementary Figure S2).

In this analysis, a trend towards high levels of SRSF1 expression in tumour samples was found to be associated with better OS in both BRCA (n = 943; HR = 0.77; log-rank *p* = 0.056; Supplementary Figure S2A, left) and COAD (n = 814; HR = 0.84; log-rank *p* = 0.16; Supplementary Figure S2A, right) patient cohorts despite the statistical insignificance. Kaplan-Meier survival analysis further demonstrated that both BRCA (n = 2032; HR = 0.64; log-rank *p* = 5.5e-09; Supplementary Figure S2B, left) and COAD (n = 1167; HR = 0.66; log-rank *p* = 0.00045; Supplementary Figure S2B, right) cohorts exhibited significantly longer DFS in patients with high-SRSF1 expression, as compared with those with low-SRSF1 expression. BRCA patients in the SRPK1-high expression tumour group, by contrast, exhibited the opposite prognostic effect on DFS (high SRPK1: n = 2473, low SRPK1: n = 2456; HR = 1.66; log-rank *p* < 1E-16; Figure 5B, left) to that associated with high SRSF1 expression; COAD patients in the SRPK1-high expression tumour group exhibited the same prognostic effect on DFS as that associated with high SRSF1 expression (high SRPK1: n = 926, low SRPK1: n = 410; HR = 0.52; log-rank *p* = 1.1e-09; Figure 5B, right). These results suggested that changes in SRSF1 expression levels may be at least partially associated with patient outcomes, as a crucial fraction of this association reflected a cancer-type-specific manner, thereby highlighting the heterogeneity across cancer types.

Taken together, these results suggested that SRPK1-driven expression of SRSF1 (i.e., their co-expression) may act in a tumour-type-dependent fashion for the analysed cancer types. As abnormal expression or activity of splicing factors is known to promote the likelihood of either exon skipping or inclusion in AS, this cooperation potentially leads to even higher levels of an oncogenic isoform; thus promoting tumour maintenance and treatment resistance-associated processes across multiple cancer types. Supplementary Figure S3 presents the schematic representation of the proposed model of action of the SRPK1-SRSF1 co-expression axis.

### 3.5 Pharmacogenomics insights on SRPK1 expression-driven sensitivity and resistance to anti-cancer agents

It has long been suggested that cancer-specific responses to anti-cancer agents may be dependent on changes in gene expression (Shea et al., 2025; Roy et al., 2019). Gene expression changes in splicing regulatory elements and/or splicing factors, both individually and in coordination, have been shown to contribute to the frequent changes in the splicing patterns of target genes across cancers and cell types, which may ultimately lead to divergent responses to anti-cancer agents (Deng et al., 2021; Anczukow et al., 2024). Findings on the alteration of SRPK1 in a cancer-specific manner and its association with poor patient prognosis and expression of distinct splicing isoforms prompted this study to assess whether changes in SRPK1 expression levels may be among the unknown driving forces behind the drug-resistance mechanisms. Therefore, this study utilised independent pharmacogenomics datasets to determine the extent of and how the relationships between changes in SRPK1 expression and responses to various anti-cancer agents in both cancer cell lines and patients’ tumour samples.

Based on the independent drug sensitivity datasets from the GDSC and CTRP, a systematic analysis was conducted to assess the influence of SRPK1 expression on the response of individual cell lines to targeted agents. This analysis included 1,001 cancer cell lines and 251 unique anti-cancer agents from the GDSC dataset and 960 cancer cell lines and 481 unique anti-cancer agents from the CTRP dataset. Cancer cell lines in both datasets are of various lineages, including breast, ovarian, colon, skin, blood, and prostate. Additionally, the anti-cancer agents target a broad range of cancer-related processes, including phosphonisitide 3-kinase (PI3K)/mammalian target of rapamycin (mTOR) signalling, extracellular signal-regulated kinase 1 (ERK)/mitogen activated protein kinase (MAPK) signalling, receptor tyrosine kinase (RTK) signalling, epidermal growth factor receptor (EGFR) signalling, apoptosis pathway, EMT, DNA damage response, and cell cycle.

Given the various tissue-specific lineages and the diversity of anti-cancer agent targets available in either dataset, an evidence-based approach was adopted, in which drug sensitivity data provides meaningful insights on the gene expression-based dependencies of drug responses. The effect of each anti-cancer agent on gene expression-based cell line sensitivity was assessed based on the IC_50_ value (drug concentration at 50% viability) from the GDSC dataset and the AUC value (area under the dose-response curve) from the CTRP dataset (where IC50 values were not reported).

As shown in Figure 8A, the analysis of the GDSC dataset for hundreds of cancer cell lines revealed the association of SRPK1-high levels with either sensitivity or resistance to the top 30 important anti-cancer agents (out of 251 tested anti-cancer agents). Of these, nine anti-cancer agents were positively correlated with SRPK1-high levels, suggesting that overexpression of SRPK1 may render multiple cancer cell lines more resistant to these agents. Conversely, 21 anti-cancer agents were negatively correlated with SRPK1-high levels, indicating that increased SRPK1 levels may enhance the sensitivity of diverse cancer cell lines to these agents. However, the relatively weak correlations between SRPK1 overexpression and alterations in response to all 30 anti-cancer agents (Pearson’s correlation coefficient *r* ranging from −0.25 to 0.25; Supplementary Table S3) were observed despite being statistically significant (FDR ≤0.05). Notably, among these thirty anti-cancer agents tested across cancer-specific cell lines, SRPK1 expression levels were most strongly and positively correlated with resistance to TGX221 (*r* = 0.243; FDR = 2.05E-05), followed by erlotinib (*r* = 0.230; FDR = 0.000246) and lapatinib (*r* = 0.214; FDR = 0.000207), and most strongly and negatively correlated with sensitivity to NPK76-II-72–1 (*r* = −0.266; FDR = 1.87E-15), PHA-793887 (*r* = −0.227; FDR = 1.9E-11), and AT-7519 (*r* = −0.209; FDR = 9.41E-10). Nonetheless, several observed anti-cancer agents have been reported to exhibit treatment responses associated with changes in gene expression levels, such as erlotinib (Lv et al., 2019; Pal et al., 2021; Yamasaki et al., 2007) and lapatinib (Jiang et al., 2018; Steggall et al., 2025; Goel et al., 2016; Ruprecht et al., 2017), as well as novel anti-cancer agents whose associations are yet to be clearly identified, including the top-ranked PHA-793887 and AT-7519. However, none of these anti-cancer agents have been linked to SRPK1.

**FIGURE 8:**
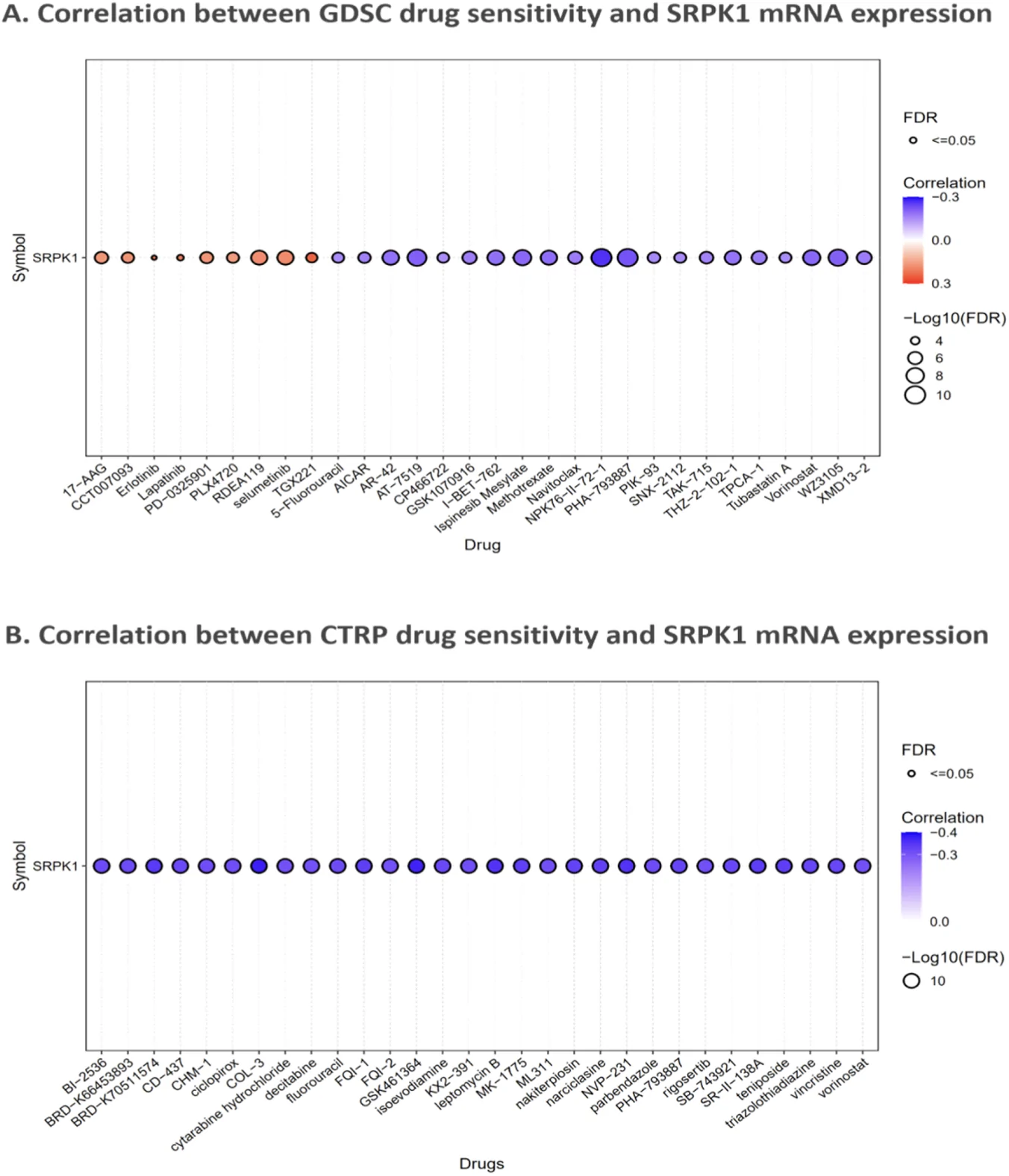
SRPK1 mRNA expression in relation to anti-cancer drug sensitivity across multiple cancer cell lines based on two independent pharmacogenomics datasets **(A)** Bubble plot representation of the top 30 agents correlated (either positively or negatively) with SRPK1 mRNA expression across 1,001 cancer cell lines in the GDSC drug sensitivity dataset, comprising a total of 251 anti-cancer agents **(B)** Bubble plot depicting the negative correlations of the top 30 agents associated with SRPK1 mRNA expression across 960 cancer cell lines in the CTRP drug sensitivity dataset, comprising a total of 481 anti-cancer agents. The correlation coefficients were calculated using Pearson’s correlation. The corresponding FDR values indicate the statistical significance of the correlations. The gradient colour bar shows the correlation coefficient; bubble size indicates the significance of the correlation, reflected by −log_10_ FDR. The bubble colour intensity signifies the degree of association with anti-cancer agent sensitivity and resistance. Red bubbles indicate a positive correlation (i.e., increased resistance to the specified agent in cancer cell lines with higher SRPK1 expression), and blue bubbles represent a negative correlation (i.e., increased sensitivity to the specified agent in cancer cell lines with higher SRPK1 expression).

Decreased H19 expression has been shown to upregulate vimentin and downregulate E-cadherin concurrently, leading to EMT and subsequent resistance to the EGFR inhibitor erlotinib in the EGFR-mutated non-small-cell lung cancer (NSCLC) cell line, HCC827 (Chen et al., 2020). In another NSCLC cell line, A549, erlotinib-induced resistance has been associated with H19-high levels, which promote the expression of ATG7 by inhibiting miR-615–3p, thereby increasing the likelihood of autophagy activation (Pan and Zhou, 2020). Together, these studies suggest that sensitivity or resistance to anti-cancer agents is likely modulated by tissue- and lineage-specific changes in gene expression. Considering the aforementioned lineage-specific findings, this may explain why no strong correlations between sensitivity/resistance and SRPK1 expression levels were observed. A possible explanation for our GDSC observation could be that pan-cancer associations, with a large and heterogeneous collection of cell lines, dilute out the strength of cancer-specific correlations that might arise due to lineage-specific differences in SRPK1 expression levels, despite these correlations being statistically significant (FDR ≤0.05). This highlights the utility of using a large and heterogeneous collection of cell lines to increase statistical power; however, it may still be insufficient to assess the contribution of gene expression levels in identifying clinically meaningful gene-drug correlations. Taken together, this indicates that sensitivity or resistance to many anti-cancer agents may be modulated by lineage-specific gene expression changes in the context of a cancer-specific analysis.

We subsequently examined whether differences in drug response are associated with changes in SRPK1 expression levels using an independent drug-sensitivity CTRP dataset. Analysis of the CTRP dataset for cancer cell lines revealed that SRPK1-high levels were associated with sensitivity to the top thirty anti-cancer agents (out of 481 tested anti-cancer agents), as shown in Figure 8B. We examined this in more detail; all thirty anti-cancer agents showed negative correlations with SRPK1-high levels, indicating that increased SRPK1 levels may enhance the sensitivity of diverse cancer cell lines to these agents. Specifically, significant (FDR ≤0.05) yet moderate negative correlations (Pearson correlation coefficient *r* ranging from −0.31 to −0.38; Supplementary Table S4) between SRPK1 expression levels and increased sensitivity to all thirty anti-cancer agents were observed. Among the thirty anti-cancer agents with negative correlations in the CTRP dataset, in cancer-specific cell lines, SRPK1 expression levels were most strongly correlated with sensitivity to COL-3 (a matrix metalloproteinase inhibitor; *r* = −0.38; FDR = 2.16E-18), followed by GSK461364 (a PLK1 inhibitor; *r* = −0.37; FDR = 5.27E-25) and NVP-231 (a selective ceramide kinase inhibitor; *r* = −0.35; FDR = 4.62E-22). In addition to the top-ranked anti-cancer agents, several other agents observed in the CTRP dataset, including BRD-K70511574 (PLK1 inhibitor; *r* = −0.34; FDR = 3.59E-21), FQI-1 (factor quinolinone inhibitor 1; *r* = −0.34; FDR = 9.14E-11), MK-1775 (Wee1 kinase inhibitor; *r* = −0.34; FDR = 9.85E-20), and SR-II-138A (translation inhibitor; *r* = −0.34; FDR = 2.05E-21), have not yet been reported to be associated with gene expression changes that are likely to play a role in differences in drug response. This observation presumably reflects the limited availability of experimental data linking gene expression to drug response for these agents. Furthermore, all thirty anti-cancer agents have not previously been linked to SRPK1; a similar pattern was observed in the GDSC dataset. Collectively, our observations not only hint at a possible link between SRPK1 expression levels and drug sensitivity or resistance but also suggest that sensitivity or resistance to anti-cancer cell lines with reasonable accuracy and reproducibility, particularly in the context of lineage-specific associations.

We next wished to move beyond cell lines to predict and interpret patient responses to therapies from gene expression using data from the CPADS platform. To do so, we accessed the CPADS platform, which features a large compendium of omic datasets (from the TCGA and GDSC databases) spanning multiple cancer types, with gene expression profiles of mostly primary patient tumours and clinical response data in the form of predicted IC_50_ values for commonly administered drugs. To examine whether differences in drug response are associated with changes in SRPK1 levels, we evaluated predicted responses to both approved and experimental anti-cancer agents, focusing especially on BRCA, COAD, and PRAD patient cohorts using gene expression data obtained from the TCGA dataset. More specifically, our analysis focused on clinically relevant chemotherapeutic agents that elicited a spectrum of differential responses: cisplatin (a DNA-damaging agent) in the BRCA and COAD patient cohorts, and docetaxel (a microtubule-targeting agent) in the PRAD patient cohort. For the examined chemotherapeutic agents, cisplatin and docetaxel, previous studies have shown that although cisplatin is widely used in treatment of patients with BRCA (Qi et al., 2023b) and COAD (Chen P. et al., 2021), and docetaxel is a standard therapy for patients with PRAD (Cevatemre et al., 2024; Domingo-Domenech et al., 2012), the acquisition of chemotherapy resistance to both agents is a devastating and widespread phenomenon in clinical oncology. Before evaluating predicted drug responses across the analysed cancer types, we stratified tumour samples into SRPK1 high- and low-expression groups and then assessed the difference in the mean of predicted IC_50_ values in the SRPK1-high *versus* SRPK1-low expression groups, along with the associated p values calculated using the two-sided Wilcoxon rank-sum test (Figure 9). A predicted drug response analysis revealed that COAD patients in the SRPK1-high expression tumour group exhibited significantly lower (predicted) IC_50_ values compared to the SRPK1-low expression tumour group for cisplatin (two-sided Wilcoxon rank-sum test; P = 0.0065; Figure 9B, middle), suggesting enhanced sensitivity to this chemotherapeutic agent. BRCA patients in the SRPK1-high expression tumour group, by contrast, exhibited higher (predicted) IC_50_ values than those in the SRPK1-low expression tumour group; however, the difference did not reach statistical significance (two-sided Wilcoxon rank-sum test; *P* = 0.064; Figure 9A, left). In the case of docetaxel, PRAD patients in the SRPK1-high expression tumour group exhibited significantly higher (predicted) IC_50_ values of docetaxel than those in the SRPK1-low expression group (two-sided Wilcoxon rank-sum test; *P* = 0.018; Figure 9C, right), suggesting that SRPK1 upregulation is likely to render resistance to docetaxel.

**FIGURE 9:**
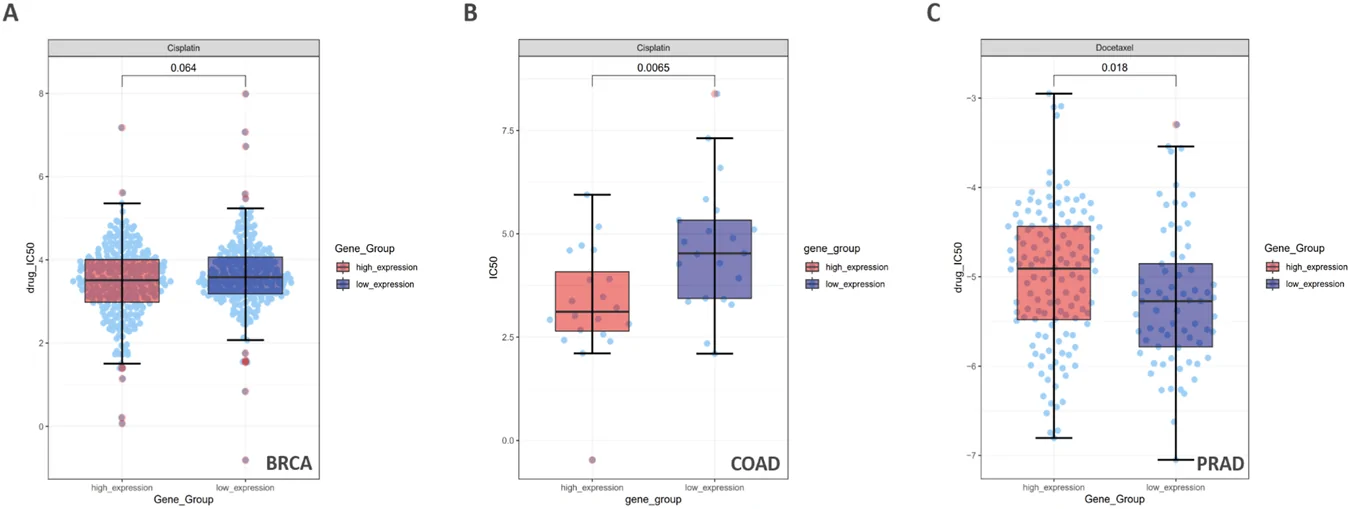
SRPK1 expression may predict sensitivity to chemotherapeutic agents in BRCA, COAD, and PRAD patient samples. Boxplots exhibit comparison of the predicted IC_50_ values of two chemotherapeutic agents, Cisplatin and Docetaxel, with SRPK1 mRNA expression, divided into SRPK1-high and SRPK1-low groups on the basis of drug sensitivity prediction from cancer-specific pharmacogenomics data of SRPK1 mRNA in tumour samples from the **(A)** TCGA-BRCA **(B)** TCGA-COAD, and **(C)** TCGA-PRAD datasets analysed using the CPADS platform. The x-axis represents SRPK1 expression groups. The y-axis represents the predicted IC_50_ values of the chemotherapeutic agent within each of the groups. The colours on the legend boxes next to the plot represent high-SRPK1 expression ( **light red** ) and low-SRPK1 expression ( **purple** ) groups, respectively. Each circle ( **light blue** ) depicts the predicted IC_50_ value of an individual of the tumour sample. The middle lines in the boxes are the median, the lower and upper boundaries of the boxes are the first and third quartiles, and the whiskers extend to 1.5× the interquartile range of the lower and the upper quartiles. The statistical significance of the difference was evaluated using the two-sided Wilcoxon rank-sum test to calculate the *P* value between the two expression groups, and *P*-values <0.05 were considered statistically significant. BRCA, Breast invasive carcinoma; COAD, Colon adenocarcinoma; PRAD, Prostate adenocarcinoma. CPADS, Comprehensive Pancancer Analysis of Drug Sensitivity.

Collectively, these results suggest that the development of resistance to cisplatin or docetaxel may be driven, at least in part, by changes in SRPK1 expression, indicating highly context- and cancer-specific associations.

## 4 Discussion

The complex and multifaceted challenge of chemoresistance necessitates the discovery of novel molecular targets and a deeper understanding of chemoresistance mechanisms in cancer cells. This study’s comprehensive pan-cancer analysis presented a robust, clinically-focused investigation into the role of SRPK1, a key regulator of alternative splicing, by integrating diverse multi-omics data.

Findings on the broad upregulation of SRPK1 mRNA across numerous tumour types, including significant elevation in BRCA and COAD at both mRNA and protein levels, strongly supported the context-specific relevance of SRPK1 to tumour progression. This was further substantiated by a prognostic analysis using the Kaplan-Meier Plotter database, which indicated the significant association of high SRPK1 expression, particularly in BRCA, with poor OS and DFS. In the context of cancer progression, genes associated with poor patient prognosis are often considered pivotal drivers and high-priority therapeutic targets due to their involvement in tumour-specific dependencies.

The functional association analysis, specifically the focus on the splicing factor SRSF1, provided a mechanistic link to the pathogenesis of chemoresistance. SRPK1 phosphorylates SR proteins like SRSF1, thereby regulating their activity and the downstream process of alternative splicing. Given that aberrant AS is an established mechanism of drug resistance—where changes in splicing factor abundance promote malignant progression and chemoresistance—the co-expression network of SRPK1 and SRSF1 suggests this pathway as a key player in tumour-specific malignancy. For instance, the AS of the pyruvate kinase gene (PKM) into its PKM2 isoform, regulated by the splicing factor PTBP1, has been associated with poor prognosis and drug resistance in pancreatic cancer (Calabretta et al., 2015), a parallel that highlights the significance of the SRPK1-SRSF1 axis in other cancers.

This study’s pharmacogenomics analysis was deemed crucial for translational relevance. The observed correlation between high SRPK1 expression and predicted resistance or sensitivity to agents like cisplatin and docetaxel in tumour cohorts provided strong clinical justification for targeting SRPK1. Findings reconciled the seemingly contradictory literature on SRPK1’s role in chemosensitivity by suggesting a tumour-specific and context-dependent modulation of the protein. For instance, while some studies found that SRPK1 downregulation restored cisplatin sensitivity (Nikas et al., 2019), others suggested a link between reduced expression and enhanced resistance (Schenk et al., 2001). The adopted multi-omics approach in this study—by defining the expression-to-prognosis-to-drug-response axis in a pan-cancer manner—introduced a more defined landscape where SRPK1-mediated effects are likely dependent on the specific tumour type and its core regulatory networks.

These opposing findings in the literature—wherein two independent studies targeting the same kinase with the same chemotherapeutic agent in ovarian carcinoma cell lines arrived at diametrically opposite outcomes regarding SRPK1’s contribution to cisplatin sensitivity (Nikas et al., 2019) —are instead an early indication of the context-dependent, dual regulatory role of SRPK1, wherein divergent baseline expression levels across distinct cell line backgrounds are sufficient to invert the functional outcome of SRPK1 activity on chemotherapy sensitivity. Whilst this dual role has begun to emerge in the literature—most notably through the demonstration that SRPK1 acetylation modulates alternative splicing to regulate cisplatin resistance in breast cancer cells (Huang et al., 2023) —the upstream regulatory contexts that determine whether SRPK1 functions as a pro-sensitivity or pro-resistance factor remain incompletely characterised across cancer types.

Critically, what remains absent across these and subsequent mechanistic studies alike is a causal account of the downstream splicing events through which SRPK1 kinase activity directly drives chemotherapy resistance: whilst the association between SRPK1 expression and drug response is increasingly well-established, the mechanistic chain linking SRPK1 activity to specific splicing factor phosphorylation—most notably of SRSF1, alongside other STRING-identified SR-family partners including SRSF2, SRSF3, SRSF4, SRSF5, SRSF6, SRSF7 and SRSF9—isoform switching at the level of individual splicing targets, and the resultant changes in chemotherapy sensitivity, has not been systematically defined across tumour types. The pharmacogenomic evidence presented in this study begins to extend this complexity to the pan-cancer level: in COAD patient cohorts, high SRPK1 expression was significantly associated with enhanced predicted sensitivity to cisplatin (*P* = 0.0065), whereas in PRAD, high SRPK1 expression was associated with predicted resistance to docetaxel (*P* = 0.018). These opposing directional associations across distinct tumour types further substantiate the dual regulatory nature of SRPK1, whilst simultaneously underscoring that pan-cancer bioinformatic approaches—however comprehensive—cannot distinguish correlation from causation, nor resolve the cell-line-specific splicing events through which SRPK1 dysregulation actively drives chemotherapy resistance. These limitations are not incidental; they reflect a fundamental boundary between associative and mechanistic evidence. It is only through the deliberate construction of acquired chemotherapy resistance in defined *in vitro* cell line models—where SRPK1 expression levels, splicing factor phosphorylation states, and downstream isoform outputs can be directly manipulated, measured, and causally linked to drug response phenotypes—that the mechanistic basis of SRPK1-driven chemoresistance can be established with sufficient precision to identify the specific splicing events that constitute actionable targets for therapeutic re-sensitisation. Establishing such causal relationships is not merely a mechanistic exercise; it carries direct clinical significance, as the identification of the downstream splicing targets through which SRPK1 dysregulation drives resistance provides the molecular rationale for pharmacological intervention—whether through SRPK1 inhibition, splice-switching strategies, or the restoration of drug uptake and apoptotic sensitivity at the level of individual isoform outputs. Such experimental models, therefore, do not merely complement bioinformatic analysis; they are the essential translational bridge between population-level expression associations and the causal, target-level clarity required to rationally re-sensitise resistant tumours to chemotherapy in a clinically meaningful and tumour-type-specific manner.

In conclusion, the multi-omics evidence presented in this study collectively positions SRPK1 as a context-dependent, dual regulatory candidate in chemotherapy response—a complexity reflected in both the opposing findings in the existing literature and the divergent pharmacogenomic associations observed here across distinct tumour types. Whilst the pan-cancer expression, prognostic, and predicted drug-response landscape of SRPK1 has been systematically characterised, the causal mechanistic chain linking SRPK1 kinase activity through splicing factor phosphorylation to specific isoform shifts and ultimately to differential chemotherapy sensitivity demands resolution at the level of individual molecular targets. Pharmacogenomic analyses presented here identify SRPK1 expression as a candidate predictor of chemotherapy response in BRCA, COAD, and PRAD; yet whether SRPK1 dysregulation constitutes a causal driver of acquired resistance or a correlate of broader transcriptomic changes—and whether its inhibition would consistently restore or paradoxically compromise sensitivity in a tumour-type-specific context—remain unresolved. If so, should experimental models be used to characterise SRPK1 activity and SRPK1-regulated splicing profiles in resistant tumours prior to SRPK1-directed therapeutic intervention? Such questions will be important to address in the context of emerging SRPK1-targeting strategies for chemotherapy-resistant breast, colon, and prostate cancers.

### 4.1 Strength of the study

By employing a comprehensive multi-omics strategy integrated with independent databases, we obtained clinically meaningful insights into alterations in SRPK1 expression levels in human tumours, encompassing pan-cancer, cancer-specific, and lineage-specific associations. Compared with related works based on transcriptomic or proteomic data alone, the integration of predicted drug responses from pharmacogenomics data reveals increased meaningful tumour heterogeneity in both gene expression patterns and drug responses within and across different cancers. Using cell-line-based pharmacogenomics databases, we predicted lineage-specific gene expression changes associated with sensitivity or resistance to many anti-cancer agents. The predictability of patients’ tumour sample response data for distinct cancer types (BRCA, COAD, and PRAD) defines the linkage between tumour-intrinsic gene expression and therapeutic responses.

### 4.2 Limitations of the study

While our analysis provides comprehensive insights into the complex and multifaceted role of SRPK1 in tumour-specific chemoresistance or chemosensitivity, several limitations must be acknowledged.

A key limitation of this study is that gene expression profiles derived from bulk RNA-seq datasets of cancer cohorts can likely be dominated by extensive sample heterogeneity, both across tumour cells and within tumour microenvironments, owing to the significant infiltration of the stroma and other cell types in variable proportions within the tumour, thereby potentially masking transcriptional circuits specific to tumour cells. This biological challenge underscores the need for further research and the development of advanced RNA-seq technologies to more accurately capture the true signals driving tumorigenesis or therapeutic resistance from rare cell populations or cell types.

While aiming for the largest sample size possible, another limitation is the relatively small sample sizes in specific pan-cancer cohorts, which constrain the conclusions that can be drawn regarding the prognostic significance of SRPK1 expression status and reduce statistical power. To address this limitation, we drew upon data from the Kaplan-Meier Plotter database, which contains a larger patient dataset, to identify statistically significant associations; however, our ability to validate survival-associated gene expression patterns was restricted by insufficient cancer availability in this database. Therefore, more comprehensive validation in larger, independent patient cohorts (i.e., expanded sample sizes) across multiple cancers, particularly in patients with BRCA, COAD, and PRAD, may be required to robustly confirm our findings regarding the prognostic and therapeutic relevance of changes in SRPK1 levels.

Moreover, a related limitation is the lack of detailed treatment information within the patient cohorts included in the multi-omics survival analyses. In the survival analysis, our patient cohorts may not be fully representative of the typical treatment outcomes observed in patients with BRCA, COAD, and PRAD, likely because of heterogeneity in treatment approaches (e.g., standard first-line treatment or combination drug therapies) and other factors (e.g., the chronological order of treatments, earlier diagnosis, and medical history) during the follow-up period. This unknown treatment heterogeneity poses challenges for our ability to correlate changes in SRPK1 expression levels with overall survival (OS) and disease-free survival (DFS), and to comprehensively interpret the survival outcomes. Based on this limitation, we conclude that integrating complementary information available in both molecular data and clinical variables represents an important approach for a better understanding of gene expression changes associated with patient outcomes, as survival analyses without treatment information may miss important disease associations.

Despite a broad spectrum of integrative network analyses, spanning gene-gene interactions, protein-protein interactions, and, in particular, co-expression correlations, there remains a substantial gap, as only functional insights into SRPK1 and its co-expressed partner SRSF1 have been uncovered; in other words, this approach cannot be used to establish direct and indirect causal relationships regarding how, and to what extent, they contribute to and cooperate in tumour maintenance and treatment resistance-associated processes across multiple cancer types. Specifically, another current challenge in our analysis of cancer-type-specific co-expression arises from the heterogeneity of cell types in bulk RNA-seq samples, which provides an aggregated view of gene expression that may differ considerably across cell types within a tumour tissue and may even obscure the detection of cell-type-, context-, and lineage-specific gene co-expression patterns. This once again highlights that sample heterogeneity, both across tumour cells and within tumour microenvironments, is a major factor driving expression changes in bulk transcriptomic data. Indeed, previous studies have demonstrated that a substantial fraction of gene co-expression in bulk data is likely to be driven by variation in cell-type abundance (McCall et al., 2016; Farahbod and Pavlidis, 2020; Zhang et al., 2021), which may be suggestive of lineage composition and, in turn, underscores the importance of lineage-specific associations in determining causality (Chen J. et al., 2021; Megyesfalvi et al., 2022). Further pre-clinical bench work is therefore required to explore the functional relevance of lineage-specific co-expression patterns and their respective contributions to tumour maintenance and treatment resistance-associated processes across multiple cancer types.

While our pharmacogenomics-based sensitivity approach, which leverages large-scale drug sensitivity data from GDSC, CTRP, and CPADS, provides valuable insights into the responses of cancer cell lines and patients’ tumour samples to several anti-cancer agents based solely on transcriptomic data, it does not capture the full spectrum of biological mechanisms that might influence treatment outcomes. Further investigation is required to elucidate the mechanisms underlying changes in SRPK1 levels and to determine whether these changes are directly or indirectly involved in the response to chemotherapy in cancers.

Although pharmacogenomics datasets are helpful to obtain a large sample size and increase statistical power, they remain limited in capturing robust associations between gene expression levels and responses to various anti-cancer agents, partly due to the restricted number of cell lines for particular cancer types, as we observed in the GDSC and CTRP datasets. We also acknowledge that chemoresistance is a complex phenomenon influenced by numerous factors beyond gene expression changes, including tumour heterogeneity, the tumour microenvironment, cooperating molecular pathways, proteomic changes, epigenetic modifications, and various clinical variables. Notably, tumour heterogeneity often leads to substantial differences in the response to anti-cancer agents among different cancer cell lines, even for the same agents, and may render the detection of AS events difficult (Urbanski et al., 2022). Therefore, in diverse cancer cell lines, more comprehensive future experimental studies are required to determine whether lineage- and context-specific changes in SRPK1 levels would likely cause, either directly or indirectly, AS deregulation involved in tumorigenesis and chemoresistance, and to investigate whether such changes would also affect cell proliferation, migration, and invasion.

## 5 Conclusion

Multi-omics approaches have rapidly developed over recent years, enabling systematic exploration of the molecular landscapes of cancer and elucidating novel aspects of cancer biology, ultimately offering a framework that could provide potential links and insights to reshape cancer research. Within this framework, the present study provides valuable multi-omics insights into how changes in SRPK1 expression may contribute to tumour maintenance and treatment resistance by comprehensively elucidating its prognostic implications, functional interaction networks, and predicted drug sensitivity across multiple cancer types, with a particular focus on BRCA, COAD, and PRAD. Taken together, our multi-omics data indicate that changes in SRPK1 levels may, at least in part, be associated with tumour-specific chemoresistance with potential relevance for poor patient outcomes in specific cancer types, thereby supporting further investigation of SRPK1-linked alternative-splicing pathways as candidate contributors to tumour-specific chemoresistance. However, as these findings are derived from retrospective analyses of bulk omics data without prospective clinical validation, integrative multi-omics data approaches alone are not sufficient to establish direct or indirect causal relationships regarding how, and to what extent, tumour-specific SRPK1 alterations contribute to tumour maintenance and/or chemoresistance. Considering all that has been discussed, our future studies would prioritise cancer cell lines, a crucial experimental tool, to further unravel tumour heterogeneity and to delve in depth into whether tumour- and context-specific SRPK1 alterations contribute to chemoresistance.

## Data Availability

The original contributions presented in the study are included in the article/Supplementary Material, further inquiries can be directed to the corresponding author.
