# Supplementary material for: Pan-cancer multi-omics profiling of SRPK1 identifies tumour-specific associations with prognosis and predicted chemotherapy response in breast, colon and prostate cancer
Source: Front Bioinform. 2026 Oct 21;6:1925354. doi: 10.3389/fbinf.2026.1925354 (PMC13731338; doi:10.3389/fbinf.2026.1925354)
Supplement: Supplementary file 1 [file DataSheet1.docx]

**Appendix 1: Supplemental Information**

**Supplementary Table S1. SRPK1 exhibits frequent differential expression across TCGA tumours, with alterations classified as up (upregulation), down (downregulation), or n.s. (not significant) based on gene expression levels.**

| **Cancer type** |  | **Disease full name** | **SRPK1 mRNA expression in cancer** |
| --- | --- | --- | --- |
| BLCA |  | Bladder urothelial carcinoma | Up |
| BRCA |  | Breast invasive carcinoma | Up |
| CESC |  | Cervical squamous cell carcinoma and endocervical carcinoma | Up |
| CHOL |  | Cholangiocarcinoma | Up |
| COAD |  | Colon adenocarcinoma | Up |
| ESCA |  | Esophageal carcinoma | Up |
| HNSC |  | Head and neck squamous cell carcinoma | Up |
| LIHC |  | Liver hepatocellular carcinoma | Up |
| LUAD |  | Lung adenocarcinoma | Up |
| LUSC |  | Lung squamous cell carcinoma | Up |
| READ |  | Rectum adenocarcinoma | Up |
| STAD |  | Stomach adenocarcinoma | Up |
| UCEC |  | Uterine corpus endometrial carcinoma | Up |
| KICH |  | Kidney chromophobe | Down |
| KIRC |  | Kidney renal clear cell carcinoma | Down |
| GBM |  | Glioblastoma multiforme | n.s. |
| KIRP |  | Kidney renal papillary cell carcinoma | n.s. |
| PAAD |  | Pancreatic adenocarcinoma | n.s. |
| PRAD |  | Prostate adenocarcinoma | n.s. |
| THCA |  | Thyroid carcinoma | n.s. |

BRCA, COAD, and PRAD are highlighted in red.

**Supplementary Table S2. The description of SRPK1-related top 28 genes identified by analysing GeneMANIA and STRING databases (Related to Figure 6).**

| **Gene** | **Full Name** |  | **Source (NCBI)** |  | **Database** |
| --- | --- | --- | --- | --- | --- |
| DDX23 | DEAD-box helicase 23 |  | https://www.ncbi.nlm.nih.gov/gene/9416 |  | STRING |
| GBP2 | Guanylate binding protein 2 |  | https://www.ncbi.nlm.nih.gov/gene/2634 |  | GeneMANIA |
| GTF3C4 | General transcription factor IIIC subunit 4 |  | https://www.ncbi.nlm.nih.gov/gene/9329 |  | GeneMANIA |
| HECW2 | HECT, C2, and WW domain containing E3 ubiquitin protein ligase 2 |  | https://www.ncbi.nlm.nih.gov/gene/57520 |  | GeneMANIA |
| LBR | Lamin B receptor |  | https://www.ncbi.nlm.nih.gov/gene/3930 |  | GeneMANIA |
| NAT10 | N-acetyltransferase 10 |  | https://www.ncbi.nlm.nih.gov/gene/55226 |  | GeneMANIA |
| PPM1G | Protein phosphatase, Mg2+/Mn2+ dependent 1G |  | https://www.ncbi.nlm.nih.gov/gene/5496 |  | GeneMANIA |
| PRM1 | Protamine 1 |  | https://www.ncbi.nlm.nih.gov/gene/5619 |  | GeneMANIA |
| PRPF38A | Pre-mRNA processing factor 38A |  | https://www.ncbi.nlm.nih.gov/gene/84950 |  | GeneMANIA |
| PRPF8 | Pre-mRNA processing factor 8 |  | https://www.ncbi.nlm.nih.gov/gene/10594 |  | GeneMANIA |
| RBM4 | RNA binding motif protein 4 |  | https://www.ncbi.nlm.nih.gov/gene/5936 |  | GeneMANIA |
| SAFB | Scaffold attachment factor B |  | https://www.ncbi.nlm.nih.gov/gene/6294 |  | GeneMANIA |
| SAFB2 | Scaffold attachment factor B2 |  | https://www.ncbi.nlm.nih.gov/gene/9667 |  | GeneMANIA |
| SERPINB10 | Serpin family B member 10 |  | https://www.ncbi.nlm.nih.gov/gene/5273 |  | GeneMANIA |
| SF3A3 | Splicing factor 3a subunit 3 |  | https://www.ncbi.nlm.nih.gov/gene/10946 |  | GeneMANIA |
| SIRT7 | Sirtuin 7 |  | https://www.ncbi.nlm.nih.gov/gene/51547 |  | GeneMANIA |
| SNRNP70 | Small nuclear ribonucleoprotein U1 subunit 70 |  | https://www.ncbi.nlm.nih.gov/gene/6625 |  | STRING |
| SRSF1 | Serine and arginine rich splicing factor 1 |  | https://www.ncbi.nlm.nih.gov/gene/6426 |  | GeneMANIA  STRING |
| SRSF2 | Serine and arginine rich splicing factor 2 |  | https://www.ncbi.nlm.nih.gov/gene/6427 |  | STRING |
| SRSF3 | Serine and arginine rich splicing factor 3 |  | https://www.ncbi.nlm.nih.gov/gene/6428 |  | STRING |
| SRSF4 | Serine and arginine rich splicing factor 4 |  | https://www.ncbi.nlm.nih.gov/gene/6429 |  | STRING |
| SRSF5 | Serine and arginine rich splicing factor 5 |  | https://www.ncbi.nlm.nih.gov/gene/6430 |  | STRING |
| SRSF6 | Serine and arginine rich splicing factor 6 |  | https://www.ncbi.nlm.nih.gov/gene/6431 |  | GeneMANIA  STRING |
| SRSF7 | Serine and arginine rich splicing factor 7 |  | https://www.ncbi.nlm.nih.gov/gene/6432 |  | STRING |
| SRSF9 | Serine and arginine rich splicing factor 9 |  | https://www.ncbi.nlm.nih.gov/gene/8683 |  | STRING |
| TARDBP | TAR DNA binding protein |  | https://www.ncbi.nlm.nih.gov/gene/23435 |  | GeneMANIA |
| ZRSR2 | Zinc finger CCH-type binding motif and serine/arginine rich 2 |  | https://www.ncbi.nlm.nih.gov/gene/8233 |  | GeneMANIA |

NCBI denotes National Centre for Biotechnology Information; GeneMANIA denotes Gene Multiple Association Network Integration Algorithm; STRING denotes Search Tool for the Retrieval of Integrating Proteins. SRSF1 is highlighted in red.

**Supplementary Fig. S1. Prognostic implications of the selected target gene SRSF1 across BRCA, COAD, and PRAD clinical cohorts (Related to Figure 6). (A)** OS and **(B)** DFS analysed in BRCA, COAD, and PRAD patient cohorts using the GEPIA2 database. Patients in the indicated cancer cohorts were stratified based on the median cutoff point of SRSF1 mRNA expression to generate the Kaplan-Meier curves of OS and DFS (plotted in months). The **red** and **blue** lines represent the survival curves of patients with high and low levels of SRSF1 mRNA. Indicated by dashed lines, HR values (with 95% confidence intervals) represent the relative risk of the low-expression group compared with the high-expression group. HR > 1 implies the gene as a potential risk factor, whereas HR < 1 suggests a protective factor. Based on a log-rank (Mantel-Cox) test, a log-rank p < 0.05 indicates statistical significance. n indicates the patient count in each expression group.

**Supplementary Fig. S2. Prognostic relevance of the selected target gene SRSF1 in BRCA and COAD patients (Related to Figure 7).** Kaplan-Meier plots revealed differences in **(A)** OS and **(B)** DFS of BRCA and COAD patients based on the 226419_s_at probe, obtained from the Kaplan-Meier plotter database. The OS and DFS of BRCA and COAD patients stratified by low (**black** line) and high (**red** line) SRSF1 expression based on the optimal cutoff point provided by Kaplan-Meier plotter database. The OS and DFS of high- and low-expression groups were compared based on HR (with 95% confidence intervals). Based on a log-rank (Mantel-Cox) test, a log-rank *p* < 0.05 indicates statistical significance.

**Supplementary Fig. S3. A proposed model of SRPK1-dependent SRSF1 expression that may drive alternative splicing isoform changes leading to oncogenic effects in a cancer-specific manner. (Left)** SRPK1 phosphorylates the splicing factor SRSF1 to regulate its functional activity in pre-mRNA splicing, thereby generating mRNA variants through the inclusion or skipping of exons or introns during the splicing process. **Light blue** boxes indicate exons, while a **light grey** box indicates an intron. Dotted **blue** lines connecting exons represent the skipped intron, and **red** lines represent the intron inclusion. P denotes phosphorylation. **(Middle)** Co-expression analysis between SRPK1 and SRSF1 at the mRNA level may identify cancer-specific biological roles with potentially crucial contributions to cancer progression across multiple cancer types. Independent survival analyses of SRPK1 and SRSF1 are likely to reveal crucial prognostic associations, whereby their expression changes correlate with favourable (or poor) outcomes in a cancer-specific manner. **(Right)** Co-upregulation (or co-downregulation) of SRPK1, together with SRSF1, may lead to changes in splicing isoforms, correlated with an increased probability of exon inclusion or skipping. Hence, in cancer cells, such distinct splicing isoforms may be involved in each of the steps of proliferation, invasion, metastasis, and treatment resistance, either directly or indirectly, through downstream targets. The **cyan** triangle represents increasing expression levels, and the **yellow** inverted triangle represents decreasing expression levels. Created with [**BioRender.com**](http://biorender.com/). Duzgun, D. (2026) [**https://BioRender.com/klzw4jf**](https://BioRender.com/klzw4jf).

**Supplementary Table S3. List of experimental profiled anti-cancer agents showing the top nine positive and the top 21 negative correlations between drug response and SRPK1-high expression levels across diverse cancer cell lines in the GDSC dataset (Related to Figure 8A).**

| **Anti-cancer agents** | **Correlation coefficient (*r*)** | **FDR** | **Pathway** |
| --- | --- | --- | --- |
| 17-AAG | 0.170 | 2.44E-06 | Protein stability and degradation |
| CCT007093 | 0.175 | 8.19E-06 | Cell cycle |
| Erlotinib | 0.230 | 0.000246 | EGFR signalling |
| Lapatinib | 0.214 | 0.000207 | EGFR signalling |
| PD-0325901 | 0.172 | 3.79E-06 | ERK/MAPK signalling |
| PLX4720 | 0.161 | 9.23E-06 | ERK/MAPK signalling |
| RDEA119 | 0.185 | 7.66E-08 | ERK/MAPK signalling |
| Selumetinib | 0.185 | 7.78E-08 | ERK/MAPK signalling |
| TGX221 | 0.243 | 2.05E-05 | PIK3/mTOR signalling |
| 5-Fluorouracil | − 0.154 | 1.18E-05 | Other |
| AICAR | − 0.168 | 7.06E-06 | Metabolism |
| AR-42 | − 0.189 | 4.69E-08 | Chromatin histone acetylation |
| AT-7519 | − 0.209 | 9.41E-10 | Cell cycle |
| CP466722 | − 0.151 | 1.32E-05 | Genome integrity |
| GSK1070916 | − 0.172 | 9.89E-07 | Mitosis |
| I-BET-762 | − 0.188 | 3.35E-08 | Chromatin other |
| Ispinesib Mesylate | − 0.199 | 8.58E-09 | Mitosis |
| Methotrexate | − 0.193 | 5.8E-08 | DNA replication |
| Navitoclax | − 0.175 | 1.07E-06 | Apoptosis regulation |
| NPK76-II-72-1 | − 0.266 | 1.87E-15 | Cell cycle |
| PHA-793887 | − 0.227 | 1.9E-11 | Cell cycle |
| PIK-93 | − 0.156 | 5.78E-06 | PIK3/mTOR signalling |
| SNX-2112 | − 0.154 | 1.08E-05 | Protein stability and degradation |
| TAK-715 | − 0.165 | 2.1E-06 | JNK and p38 signalling |
| THZ-2-102-1 | − 0.185 | 9.58E-08 | Cell cycle |
| TPCA-1 | − 0.174 | 3.93E-07 | Kinases other |
| Tubastatin A | − 0.153 | 1.05E-05 | Chromatin histone acetylation |
| Vorinostat | − 0.201 | 1.47E-08 | Chromatin histone acetylation |
| WZ3105 | − 0.208 | 1.06E-09 | p53 signalling |
| XMD13-2 | − 0.174 | 4.82E-07 | Cytoskeleton |

R values represent Pearson’s correlation coefficients; the statistical significance of the correlation is determined based on the corresponding false discovery rate (FDR ≤ 0.05); r > 0 indicates positive correlation; r < 0 indicates negative correlation; erlotinib, lapatinib, and TGX221 are highlighted in red; AT-7519, NPK76-II-72-1, and PHA-793887 are highlighted in blue.

**Supplementary Table S4. List of experimental profiled anti-cancer agents showing the top 30 negative correlations between drug response and SRPK1-high expression levels across diverse cancer cell lines in the CTRP dataset (Related to Figure 8B).**

| **Anti-cancer agents** | **Correlation coefficient (*r*)** | **FDR** | **Pathway** |
| --- | --- | --- | --- |
| BI-2536 | − 0.32 | 4.15E-19 | Cell cycle |
| BRD-K66453893 | − 0.32 | 5.05E-18 | ERK/MAPK signalling other |
| BRD-K70511574 | − 0.34 | 3.59E-21 | Cell cycle |
| CD-437 | − 0.32 | 3.76E-18 | Apoptosis regulation other |
| CHM-1 | − 0.31 | 7.96E-18 | Mitosis |
| Ciclopirox | − 0.31 | 5.27E-18 | Chromatin histone acetylation other |
| COL-3 | − 0.38 | 2.16E-18 | Other |
| Cytarabine hydrochloride | − 0.31 | 2.41E-17 | p53 signalling other |
| Decitabine | − 0.31 | 1.66E-17 | Other |
| Fluorouracil | − 0.31 | 1.82E-17 | Metabolism other |
| FQI-1 | − 0.34 | 9.14E-11 | Other |
| FQI-2 | − 0.31 | 1.46E-17 | Other |
| GSK461364 | − 0.37 | 5.27E-25 | Cell cycle |
| Isoevodiamine | − 0.32 | 1.72E-18 | Apoptosis regulation other |
| KX2-391 | − 0.31 | 2.14E-17 | Metabolism other |
| Leptomycin B | − 0.35 | 3.36E-23 | p53 signalling |
| MK-1775 | − 0.34 | 9.85E-20 | Cell cycle |
| ML311 | − 0.31 | 6.96E-18 | Apoptosis regulation |
| Nakiterpiosin | − 0.33 | 1.74E-19 | Mitosis |
| Narciclasine | − 0.33 | 1.38E-19 | Other |
| NVP-231 | − 0.35 | 4.62E-22 | Other |
| Parbendazole | − 0.31 | 1.54E-18 | Mitosis |
| PHA-793887 | − 0.33 | 1.01E-19 | Cell cycle |
| Rigosertib | − 0.31 | 7.80E-17 | PIK3/mTOR signalling |
| SB-743921 | − 0.32 | 2.45E-19 | Mitosis |
| SR-II-138A | − 0.34 | 2.05E-21 | Other |
| Teniposide | − 0.34 | 3.94E-11 | DNA replication |
| Triazolothiadiazine | − 0.33 | 5.21E-20 | Other |
| Vincristine | − 0.32 | 4.26E-20 | Cytoskeleton |
| Vorinostat | − 0.31 | 6.17E-17 | Chromatin histone acetylation |

R values represent Pearson’s correlation coefficients; the statistical significance of the correlation is determined based on the corresponding false discovery rate (FDR ≤ 0.05); r < 0 indicates negative correlation.

**Supplementary Table S5. Methodological summary of multi-omics data resources and analytical tools used in the SRPK1 analysis workflow.**

| **Platform/Web Tool** | **Omics/Data Layer** | **Underlying Datasets/Resource** | **Analysis Performed** | **Version/Release Identifier Access Date** | **Key Settings/Parameters** | **Cohort/ Sample Stratification** | | **Random- Seed Applicability** | **Reference** |
| --- | --- | --- | --- | --- | --- | --- | --- | --- | --- |
| TIMER2 | Transcriptomics | TCGA tumour and adjacent normal tissue; RNA-seq data | Pan-cancer SRPK1 mRNA tumour-normal expression analysis | Tumor Immune Estimation Resource 2.0 (TIMER2); accessed on 25 September 2025 | Gene_DE module; Wilcoxon rank-sum test | | Not applicable | Not applicable; no stochastic procedure was performed and no user-definable seed exists | TIMER2 web resource; URL provided in the Methods section |
| GEPIA2 | Transcriptomics; Survival-associated expression analysis; Co-expression analysis | TCGA-tumour data and GTEx normal tissue data where applicable | SRPK1 mRNA expression analysis; SRPK1 survival analysis; identification of SRPK1-associated genes; SRPK1-SRSF1 co-expression analysis | GEPIA2, Gene Expression Profiling Interactive Analysis version 2; accessed on 25 September 2025 | Expression DIY; Survival Analysis; Similar Gene Detection; Correlation Analysis; median split; log-rank test; Pearson correlation | | Survival cohorts stratified using the predefined median expression cut-off: 50% high and 50% low SRPK1 expression | Not applicable; median-based stratification is deterministic and does not require a random seed | *[42]* |
| UALCAN-CPTAC | Proteomics | CPTAC proteomic datasets | SRPK1 total protein expression analysis in BRCA, COAD, and PRAD | UALCAN CPTAC protein-analysis module; the portal does not issue a version identifiers; accessed on 25 September 2025 | CPTAC protein-analysis module; tumour-normal and age-stratified comparisons | | Not applicable | Not applicable; no stochastic procedure was performed and no user-definable seed exists | *[43]* |
| HPA | Protein expression; Immunohistochemistry | HPA immunohistochemistry images and antibody annotation | Immunohistochemical validation of SRPK1 protein expression in normal and tumour tissues | Human Protein Atlas version 24; accessed on 25 September 2025 | SRPK1 antibody HPA016431 | | Not applicable | Not applicable; no stochastic procedure was performed and no user-definable seed exists | HPA web resource; URL provided in the Methods section |

**Supplementary Table S5** (Continued)

| **Platform/Web Tool** | **Omics/Data Layer** | **Underlying Datasets/Resource** | **Analysis Performed** | **Version/Release or Access-Date Information** | **Key Settings/Parameters** | **Cohort/ Sample Stratification** | **Random- Seed Applicability** | **Reference** |
| --- | --- | --- | --- | --- | --- | --- | --- | --- |
| Kaplan-Meier Plotter | Transcriptomics-linked clinical survival analysis | Kaplan-Meier Plotter survival cohorts for BRCA and COAD; corresponding PRAD data were not available in this platform | Independent survival validation for OS and RFS/DFS according to SRPK1 expression in BRCA and COAD | Kaplan-Meier Plotter web tool; accessed on 25 September 2025 | OS and RFS/DFS; Auto select best cutoff | Patients stratified using the platform-provided “Auto select best cutoff” function | Not applicable; the platform-defined cut-off procedure was used and no user-defined random seed was available or required | Kaplan-Meier Plotter web resource; URL provided in the Methods section |
| GeneMANIA | Functional genomics; Gene-network analysis | GeneMANIA functional interaction resources | SRPK1-related gene-network analysis | GeneMANIA web portal; accessed on 27 September 2025 | SRPK1 query; top 20 associated genes | Not applicable | Not applicable; no stochastic procedure was performed | *[44]* |
| STRING | Protein-protein interaction network | STRING protein-protein association database | SRPK1-interacting protein network analysis | STRING version 12.0; accessed on 27 September 2025 | Confidence edges; high confidence = 0.7; maximum 10 first-shell interactors | Not applicable | Not applicable; fixed web-interface parameters were used and no stochastic procedure was performed | *[45]* |
| GSCA | Transcriptomics; Pharmacogenomics | TCGA gene expression data; GDSC and CTRP pharmacogenomic drug-sensitivity datasets | Correlation analysis between SRPK1 mRNA expression and drug sensitivity across cancer cell lines | GSCA pharmacogenomics platform; accessed on 27 September 2025 | Drug-sensitivity module; platform-reported Pearson r and FDR-adjusted values | Not applicable | Not applicable; fixed platform-derived correlation outputs were used and no user-defined random sampling, bootstrapping, train-test partitioning or stochastic model training was performed | *[46]* |
| CPADS | Transcriptomics; Pharmacogenomics; Predicted drug response | TCGA transcriptomic data and GDSC drug-response data | Predicted patient-level chemotherapy response analysis according to SRPK1 expression | CPADS, Comprehensive Pancancer Analysis of Drug Sensitivity web tool; accessed on 27 September 2025 | Drug Analysis—By Gene; predicted log(IC_50_); Wilcoxon rank-sum test | TCGA patient samples stratified into high- and low-SRPK1 groups using the median SRPK1 expression cut-off | Not applicable; median-based stratification was deterministic and no random seed was required | *[47]* |
